# The penicillin-binding protein PBP1b fortifies the *Escherichia coli* division site against osmotic rupture

**DOI:** 10.1038/s41564-026-02403-6

**Published:** 2026-07-03

**Authors:** Paula P. Navarro, Andrea Vettiger, Roman Hajdu, Virly Y. Ananda, Alejandro López-Tavares, Ernst W. Schmid, Johannes C. Walter, Martin Loose, Luke H. Chao, Thomas G. Bernhardt

**Affiliations:** 1 Department of Fundamental Microbiology, Faculty of Biology and Medicine, University of Lausanne, Lausanne, Switzerland; 2 Department of Microbiology, Blavatnik Institute, Harvard Medical School, Boston, MA USA; 3 Department of Molecular Biology, Massachusetts General Hospital, Boston, MA USA; 4 Department of Genetics, Blavatnik Institute, Harvard Medical School, Boston, MA USA; 5 Institute of Science and Technology Austria (ISTA), Klosterneuburg, Austria; 6 Department of Biological Chemistry & Molecular Pharmacology, Blavatnik Institute, Harvard Medical School, Boston, MA USA; 7 Howard Hughes Medical Institute, Harvard Medical School, Boston, MA USA

**Keywords:** Cellular microbiology, Cell growth, Bacteriology

## Abstract

The divisome apparatus synthesizes septal peptidoglycan (PG) during bacterial division. In *Escherichia coli*, the class A penicillin-binding protein (aPBP) called PBP1b has been implicated in division, but its role in the process has remained unclear. Here we show using in situ cryo-electron tomography, genetics and other imaging methods that PBP1b is required to produce a wedge-like density of PG at the division site and that loss of this structure weakens the division site, making it hypersusceptible to osmotic lysis. Surprisingly, the activator LpoB needed for general PBP1b function was not required for its role in division. Of the two PBP1b isoforms produced in cells, we show that the one with an extended cytoplasmic N terminus localizes to and functions at the division site, probably via recruitment by the FtsA component of the divisome. The conservation of aPBPs with extended cytoplasmic N termini suggests that other Gram-negative bacteria may use similar mechanisms for division site reinforcement.

## Abstract (web summary)

A specific isoform of PBP1b functions independently of the activator protein, LpoB, to drive generation of a wedge-like peptidoglycan structure that strengthens the division site in *Escherichia coli*.

## Main

Cell division in bacteria is mediated by a multiprotein machine referred to as the divisome or septal ring^1^. This essential process is initiated by the coalescence of treadmilling polymers of the tubulin-like FtsZ protein into a dynamic ring pattern called the Z-ring at the prospective site of division^2–5^. The Z-ring is attached to the inner face of the cytoplasmic membrane via membrane-bound FtsZ interacting proteins such as FtsA^6,7^. Following Z-ring formation, a number of essential and non-essential division proteins are recruited to the division site to form the mature divisome apparatus capable of promoting cytokinesis^1^.

**Fig. 1:**
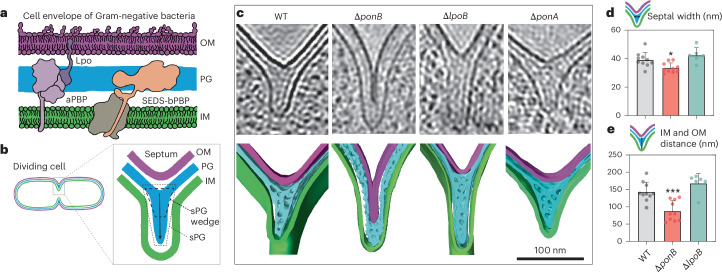
In situ septal PG architecture of wild-type and mutant *E. coli* cells. **a**, Summary diagram of the different types of bacterial PG synthase. **b**, Schematic showing the process of septum formation in Gram-negative bacteria. **c**, Top row: summed, projected central slices of low-pass filtered cryo-electron tomograms visualizing sPG architecture of the indicated *E. coli* strains. Bottom row: 3D segmentations of the cryo-electron tomograms above, rendering the IM as a green surface, the sPG as a cyan surface and the OM as a magenta surface. **d**, Bar graph showing septal width measured orthogonal to the invagination direction in cryo-ET data (mean ± s.d. for WT = 39.13 ± 5.13 nm, *∆ponB* = 33.49 ± 3.5 nm, *∆lpoB* = 42.43 ± 5.19 nm). Significant differences relative to WT were determined using one-way ANOVA with Dunnett’s post test (WT vs ∆*ponB* *P = 0.0214, WT vs *∆lpoB* *P* = 0.2997). **e**, Bar graph showing measured IM–OM distances at the septal region in the cryo-ET data. Thirty Euclidean distances were measured per data point (see Methods), *N* values for each strain indicate the number of individual measurements per side of the division site (see Methods): *N* = 10 (WT), 10 (*∆ponB*), 6 (*∆lpoB*). Significant differences relative to WT were determined using one-way ANOVA with Dunnett’s post test (WT vs ∆*ponB* ***P = 0.0005, WT vs *∆lpoB* *P* = 0.1807). Scale bar, 100 nm. Source data

A major function of the divisome is to synthesize the peptidoglycan (PG) cell wall material that will eventually fortify the poles of the daughter cells. Bacteria typically encode two main types of PG synthesis enzyme: (1) the bifunctional class A penicillin-binding proteins (aPBPs) that can both polymerize and crosslink glycans to form the PG matrix or (2) the two-component synthases formed by complexes between a SEDS-family glycan polymerase and a monofunctional class B PBP (bPBP) with PG crosslinking activity^1^ (Fig. 1a). With only one known exception^8^, the essential bacterial cell division PG synthase is a SEDS–bPBP-type enzyme composed of the FtsW (SEDS) and FtsI (bPBP) proteins^9^. aPBPs have also been implicated in cell division, but their role in the process has not been clearly defined.

*Escherichia coli* encodes two aPBPs that play important roles in PG biogenesis: PBP1a and PBP1b. These enzymes require a cognate outer membrane lipoprotein activator for their cellular activity^10,11^ (Fig. 1a). LpoA activates PBP1a whereas LpoB activates PBP1b (Fig. 1a). Neither enzyme system is essential, but their simultaneous inactivation is lethal^12,13^ and results in rapid cell lysis from lesions that develop at what appear to be random locations around the cell body as well as the division site^10,11^. Thus, aPBPs are thought to play a general role in fortifying the PG matrix, especially in areas of low PG density or where damage has occurred^11,14,15^. The rate of PG synthesis in cells harbouring PBP1b as the only aPBP is equivalent to that in WT cells and dramatically reduced when PBP1b is inactivated^14^. In addition, mutants lacking PBP1b or LpoB, but not PBP1a or LpoA, are mechanically less stiff in the cylindrical region of the cell^16^ and are hypersensitive to beta-lactams and other perturbations to PG biogenesis^12,17–19^. Thus, of the two aPBP systems, PBP1b–LpoB appears to play the predominant role in the general growth and maintenance of the PG matrix.

In addition to a general role in the expansion and fortification of the PG matrix, PBP1b is also thought to play a substantial role in cell division^20,21^. This functional assignment preceded the discovery of PG synthase activity for SEDS–bPBP complexes such as FtsW–FtsI, such that PBP1b was the only logical choice at the time. Models that propose a role for PBP1b in division are principally based on protein crosslinking and co-purification experiments that identified interactions between PBP1b and several different components of the divisome^22–26^. What has been missing is a clear demonstration that these interactions and their effects on PBP1b activity observed in vitro are physiologically relevant and important for proper division in cells. Currently, the only genetic links between PBP1b and cell division are the observations that PBP1b inactivation results in a modest reduction in PG labelling at the division site^27^ and that deletion of the *ponB* gene encoding PBP1b is synthetically lethal with the inactivation of several non-essential division factors^28–30^. However, whether these phenotypes arise due to the general defect in PG biogenesis displayed by *ponB* mutants or a specific role for PBP1b in cell division is not known.

Here we describe the discovery of a dedicated division function for PBP1b in *E. coli* enabled by our recent in situ ultrastructural analysis of *E. coli* division sites using cryo-focused ion beam (cryo-FIB) milling and cryogenic electron tomography (cryo-ET)^31^. Gram-negative bacteria such as *E. coli* have a complex cell envelope made of two membranes with a relatively thin layer of PG cell wall sandwiched in the periplasmic space between them^32^ (Fig. 1a). When they divide, the inner membrane (IM) is invaginated and new PG material is synthesized within the invagination (Fig. 1b). Constriction of the IM at the division site is often observed to be deeper than that of the outer membrane (OM)^31,33,34^. Because this invaginating structure partially bisects the daughters, it is often referred to as the division septum and the associated cell wall material is called septal PG (sPG). This PG material is initially shared between daughter cells and must be progressively split during the division process by PG cleaving enzymes to allow the outer membrane to invaginate and eventually cover the new daughter cell pole^35^.

The combination of cryo-FIB milling and cryo-ET allowed us to resolve new features of septal architecture^31^. At the leading edge of the invaginating inner membrane, two plates of density were resolved that correspond to the PG material that will form the polar cell wall of the daughters. The architecture at the lagging edge of the septum closest to the outer membrane was markedly different. This region of the septum contained a thick, wedge-like density of PG^31^. The function of this so-called ‘sPG wedge’ and the factors required for its synthesis have remained unclear. Given that genetic and imaging data point towards the FtsW–FtsI synthase functioning in sPG biogenesis at the leading edge of the invaginating septum^27,36^, we hypothesized that PBP1b might be functioning in the biogenesis of the sPG wedge. In situ cryo-ET imaging revealed that mutants lacking PBP1b indeed failed to form an observable sPG wedge. In addition, atomic force microscopy (AFM) showed that PG purified from a mutant lacking PBP1b had a reduced stiffness at the division site relative to the side wall, whereas the opposite was true for PG isolated from wild-type cells. Notably, PBP1b inactivation was shown to result in a hypersensitivity to osmotic shock with the mutant cells lysing from lesions at midcell, indicative of a compromised septum. Surprisingly, the PBP1b activator LpoB^10,11^ was not required for sPG wedge formation or the osmotic stability of the septum. Finally, we show that of the two isoforms of PBP1b known to be produced in cells^37–39^, it is the longer protein with an extended cytoplasmic N terminus that is recruited to the division site and that this recruitment is likely to be mediated by an interaction with the FtsA component of the Z-ring. Altogether, our results demonstrate that PBP1b plays a specialized, LpoB-independent role in *E. coli* cell division involving the biogenesis of an sPG wedge structure that fortifies the septum against osmotic rupture.

## Results

### PBP1b is required for sPG wedge formation

**Extended Data Fig. 1:**
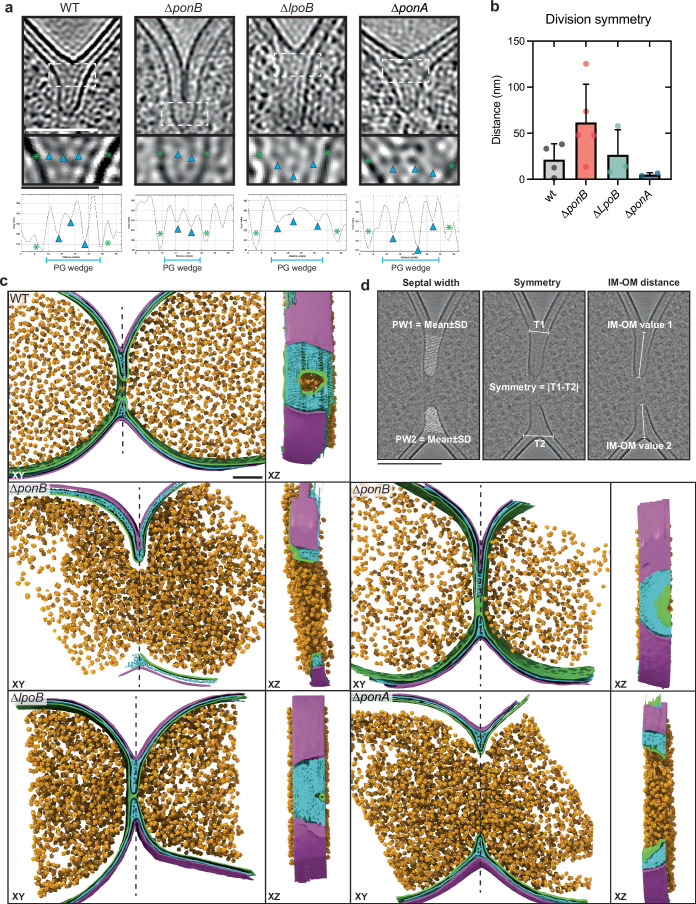
Detailed architecture of the division site by in situ cryo-ET. (**a**) Summed, projected central slices of low-passed filtered cryo-electron tomograms shown in Fig. 1. White dashed box indicates the region of corresponding zoom-in images of the typical sPG wedge-containing region below. Bottom row shows corresponding normalized grey-scale profiles of the sPG wedge region. Green asterisks indicate IM regions and blue arrows indicate PG. Note the reduced number of detectable PG densities within the septum of the ∆ponB cell. (**b**) Bar graph showing the difference in nm between the length of the tangent line to the OM tip from IM to IM of both sides of the division site. This denotes division site symmetry. (**c**) 3D surface segmentation renderings of IM (green), PG (cyan), OM (magenta) and ribosomes (yellow) are shown as top view (XY plane) and side view (XZ plane). Dashed line indicates the surface cut made to show a cross view of the PG architecture at the division site in the corresponding side views (XZ). (**d**) Example of measurements performed in Fig. 1d, e and Extended Data Fig. 1b in a summed projected central slice of dividing wild-type E.coli. N values for each strain are: N = 5 (WT); 5 (∆ponB); 3 (∆lpoB) and 2 (∆ponA). Scale bars = 100 nm. Source data

**Extended Data Fig. 2:**
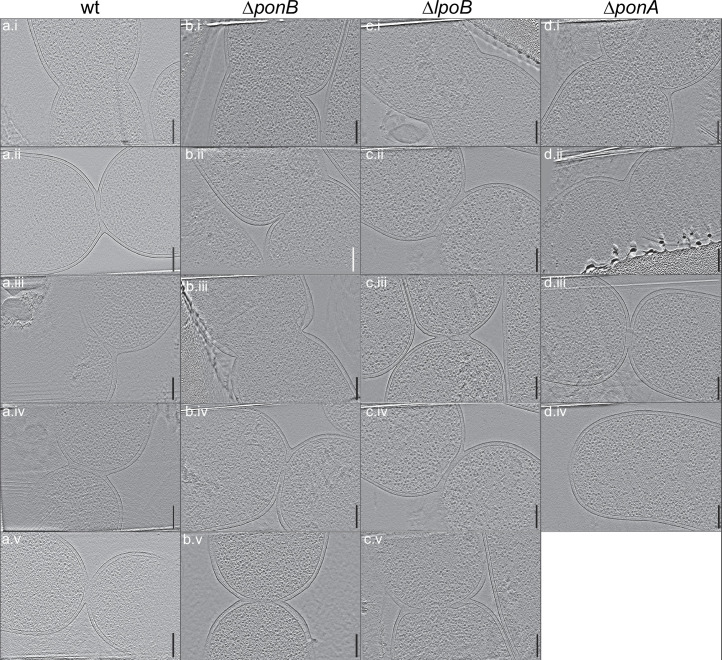
Gallery of cryo-ET data of dividing E. coli cells. Three dimensional slices visualizing the division site and pole of the indicated strains. Scale bar = 200 nm. A complete overview of number of tomograms (number of cells imaged) and data acquisition is reported in Table S1. WT data from^31^. Scale bars = 200 nm. Source data

To investigate the potential role of PBP1b in sPG wedge formation, we used in situ cryo-ET to image *E. coli* cells deleted for genes encoding PBP1b (*∆ponB*) or its lipoprotein activator LpoB (*∆lpoB*). Bacterial lamellae were generated by cryo-FIB milling, tilt series were collected and three dimensionally (3D) reconstructed into cryo-electron tomograms. The tomograms were denoised and low-pass filtered to visualize sPG architecture in situ (Supplementary Table 1). The IM, PG and OM were segmented, and oriented ribosomes were localized by template matching (see Methods and Supplementary Videos 1–4). For comparison, we used cryo-ET data of wild-type (WT) *E. coli* from our previous study^31^. Three-dimensional renderings revealed architectural differences at division sites among strains (Extended Data Fig. 1c), with representative unfiltered tomogram galleries shown in Extended Data Fig. 2. Cells lacking PBP1b exhibited slightly thinner (16.8%) septa than those of WT when measured orthogonal to membrane invagination (Fig. 1c,d and Extended Data Fig. 2). More strikingly, the OM–IM distance at the constriction was significantly reduced in *∆ponB* cells, accompanied by loss of the wedge-like sPG density separating the membranes relative to WT (Fig. 1c,e and Extended Data Fig. 2). Overall, the in situ cryo-ET results indicate that the PG density was reduced in cells lacking PBP1b, consistent with previous measurements using other methods^14,40^. No notable differences in septal architecture were observed for cells lacking PBP1a versus WT (Fig. 1c, and Extended Data Figs. 1a and 2) as expected, given that this aPBP has not previously been associated with major cell wall or division phenotypes^12,17–19^. Surprisingly, the architecture of septa in cells lacking the PBP1b activator LpoB was also largely comparable to that of WT, displaying similar width and OM–IM distance measurements (Fig. 1c–e, and Extended Data Figs. 1 and 2).

**Fig. 2:**
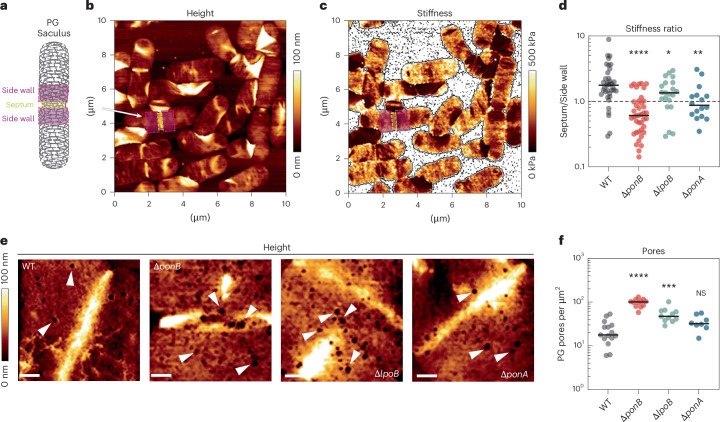
AFM analysis of PG sacculi from wild-type and mutant *E. coli* cells. PG sacculi from the indicated strains were isolated and imaged by AFM. **a**, Schematic overview of septal (yellow box) and sidewall (magenta box) regions analysed for stiffness. **b**,**c**, Representative images from (**b**) height (sample topography relative to setpoint) and (**c**) stiffness (Young’s moduli) scans of WT PG sacculi at low resolution (10 × 10 µm, 39.1 nm pixel size). Regions of analysis for septal/sidewall comparisons are highlighted with an arrow and coloured as in **a**. **d**, Ratio of septal to side wall PG stiffness was measured for the indicated strains and significant differences relative to WT were determined using one-way ANOVA with Dunnett’s post test. *P = 0.0377, **P = 0.0042, ****P = 2.9754 × 10^−7^; *N* sacculi for each strain from 3 biological replicates were measured: *N* = 37 (WT), 40 *(∆ponB*), 21 (*∆lpoB*), 15 (*∆ponA*). **e**, Representative high-magnification (1 × 1 µm, 7.81 nm pixel size) height images of septa isolated from indicated strains. Arrowheads indicate pores in the cell wall. Scale bar, 200 nm. **f**, Quantification of pores per µm^2^. Significant differences relative to WT were determined using one-way ANOVA with Dunnett’s post test. ***P = 0.0002, ****P = 1.22 × 10^−13^; NS, non-significant, *P* = 0.2655; *N* sacculi for each strain from 3 biological replicates were measured: *N* = 15 (WT), 12 *(∆ponB*), 11 (*∆lpoB*), 8 (*∆ponA*). Source data

**Extended Data Fig. 3:**
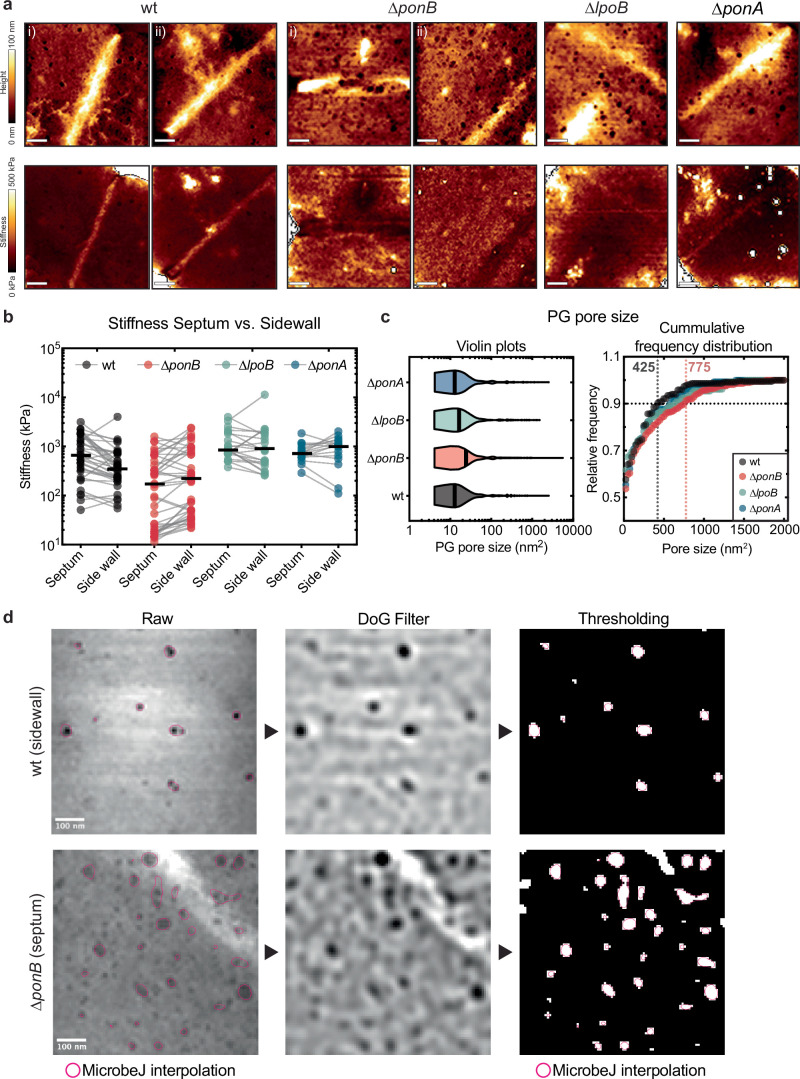
Septal ultrastructure and stiffness characterization of isolated PG sacculi by AFM. (**a**) Additional examples of high-resolution (1 × 1 µm, 7.81 nm pixel size) height (top) and Young’s modulus (stiffness, bottom) maps of indicated strains. Scale bar = 200 nm. (**b**) Absolute quantification of septal and sidewall stiffness of indicated strains. Lines connect measurements from the same sacculi. N sacculi for each strain from 3 biological replicates were measured: N = 37 (WT); 40 (∆ponB); 21 (∆lpoB); 15 (∆ponA). (**c**) Violin plots (left) and cumulative frequency distribution (right) of PG pore size as determined from high-resolution height images (see Methods). Solid line represents median pore size (violin plots), dotted lines and numbers indicates the 90th percentile of the cumulative distribution corresponding to 425 nm (WT) and 775 nm (∆ponB). In other words, 90% of the peptidoglycan pores are ≤ 425 nm2 or ≤ 775 nm2 in surface area, respectively. Numbers of pores measured for each strain were: 254 (WT); 508 (∆ponB); 144 (∆lpoB); 147 (∆ponA). (**d**) Example of image analysis procedure for quantification of PG pores. A difference of gaussian (DoG) filter was applied prior to using thresholding (default, FIJI) and interpolation in MicrobeJ (outlined in magenta).

To complement the cryo-ET imaging, we examined the properties of sPG produced by WT and mutant cells by analysing the topography and stiffness of purified PG sacculi using AFM (Fig. 2 and Extended Data Fig. 3). WT sacculi had division sites that were stiffer than sidewalls, whereas *∆ponB* sacculi showed the opposite trend, with reduced stiffness at division sites (Fig. 2d and Extended Data Fig. 3a,b). Another major difference was the frequency of lesions or ‘pores’ in the PG network observed in the AFM scans of PG topography. Cells lacking PBP1b had an increased number of pores per total PG area and the pores tended to be larger in size that those detected in WT PG (Fig. 2e,f and Extended Data Fig. 3c). This finding supports the idea that a major function of the PBP1b–LpoB system is to identify and ‘fill-in’ areas of low density within the PG matrix^11,14,15^. Accordingly, PG sacculi from *∆lpoB* cells showed a similar increase in pore frequency to those from *∆ponB* cells (Fig. 2d–f and Extended Data Fig. 3), although the sPG/side wall stiffness ratio was most significantly reduced in *∆ponB* sacculi (Fig. 2d). The sPG/side wall stiffness ratio was reduced in *∆ponB* relative to *∆lpoB* sacculi as expected but the statistical significance was marginal (*P* = 0.11), probably due to the small sample size and several outliers in the *∆lpoB* sample. Only a small reduction in the sPG/side wall stiffness ratio was observed for sacculi purified from cells lacking PBP1a (Fig. 2d). These sacculi also did not display a significant increase in the frequency of pores in the PG matrix (Fig. 2d–f and Extended Data Fig. 3). Together, the cryo-ET and AFM analyses indicate that PBP1b is required for biogenesis of the sPG wedge structure at the division site. Furthermore, this division function is not dependent on the LpoB activator, indicating that it is a specialized activity of the PG synthase distinct from its general role in PG biogenesis.

### The sPG wedge fortifies the division site against osmotic rupture

**Fig. 3:**
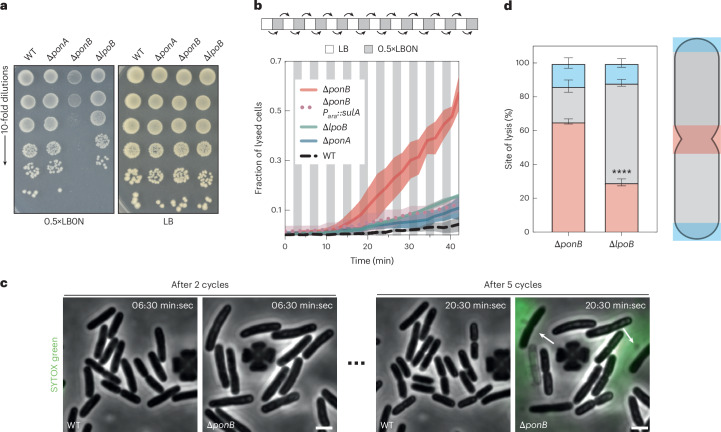
PBP1b stabilizes the *E. coli* division site against osmotic rupture. **a**, Representative image of a bacterial viability assay. Cells were grown on LB or 0.5×LB0N agar at 42 °C as indicated. **b**, Osmotic oscillations between LB (white) and 0.5×LB0N (grey) were performed using the CellAsic microfluidic platform. The fate of cells from the indicated strains was monitored at 0.1 Hz for 42 min by phase-contrast and SYTOX Green (1 µM) imaging. Filamentation was induced by expressing the division inhibitor SulA for 20 min before exposure to the first osmotic shock. SulA production was induced using 0.2% L-arabinose. Graph shows the mean fraction of lysed cells (line) ±1s.d. (shading) plotted from *N* biological replicates: *N* = 6 (WT), 5 (*∆ponB*), 4 (*∆lpoB*), 3 (*∆ponB* +SulA), 3 (*∆ponA*). **c**, Representative images of indicated strains after two and five cycles of osmotic shocks. Arrows point towards sites of septal lysis events. Scale bar, 2 µm. **d**, Manual quantification of subcellular lysis sites in response to osmotic shocks from 100 cells from 3 biological replicates. Data represented as mean ± 1s.d. and significance was tested using two-sided unpaired *t*-test. ****P = 1.2085 × 10^−5^. Source data

**Extended Data Fig. 4:**
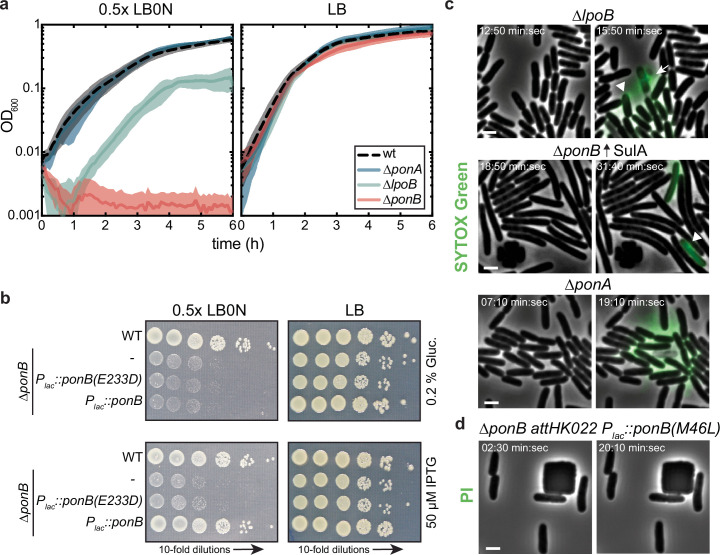
Cells lacking PBP1b are sensitive to osmotic shifts. (**a**) Growth of the indicated strains was assessed on a plate reader at 42 °C in LB or 0.5xLB0N, respectively. Day cultures of the indicated strains were back diluted to OD600 = 0.01 and growth was followed for 6 h. The line represents mean ± one SD of the absorbance value obtained from three biological replicates. (**b**) Representative image of a bacterial viability assay. Cells were grown on LB or 0.5xLB0N agar supplemented with either 0.2% glucose or 50 µM IPTG at 42 °C as indicated. Strains deleted for ponB carry indicated single-copy alleles at the attHK022 phage integration site. Data is representative for 3 biological replicates. (**c**) Representative images of additional mutants imaged in response to osmotic oscillations (see Fig. 3b). in the second set of panels filamentation was induced by expression of sulA from pNP146 for 20 min prior exposure to the first osmotic shock. White arrows point to sites of septal lysis events, and arrowheads highlight polar lysis events. (**d**) Representative image of the indicated strain quantified in Fig. 6b. Source data

We previously speculated that the sPG wedge contributes to septal osmotic stability^31^. Consistent with this hypothesis, deletion of *ponB* or inactivation of its PG polymerase activity resulted in a severe growth defect at high temperature (42 °C) in low-osmolyte medium (half-strength LB medium with no added NaCl, 0.5×LB0N) (Fig. 3a and Extended Data Fig. 4a,b). Mutants defective for LpoB or PBP1a grew normally under this condition, indicating that the phenotype is not due to general cell wall defects (Fig. 3a).

To assess whether the osmotic sensitivity reflected failure in septal integrity, we imaged cells in a microfluidic chamber during repeated osmotic shifts between normal LB and 0.5×LB0N (Fig. 3b–d and Supplementary Video 5). Imaging was performed at 37 °C where the *∆ponB* growth defect is less severe than at 42 °C. SYTOX Green was also included in the medium to stain extracellular DNA and aid the visualization of cell lysis. Over a series of 20 osmotic shifts, WT cells grew normally with minimal lysis events observed (Fig. 3b,c and Supplementary Video 5). A modest elevation in the frequency of cell lysis was observed for cells lacking LpoB or PBP1a, but lysis was dramatically increased in cells inactivated for PBP1b (Fig. 3b,c, Extended Data Fig. 4c and Supplementary Video 5). Importantly, blocking cell division by expression of the FtsZ antagonist SulA greatly reduced the level of lysis observed for *∆ponB* cells (Fig. 3b,c, Extended Data Fig. 4c and Supplementary Video 5). Moreover, when the site of cell lysis was analysed on the basis of the location of membrane bleb formation and DNA release, cells lacking PBP1b lysed from septal failures at a much higher rate than cells lacking LpoB (Fig. 3d). These results indicate that PBP1b is critical for the stabilization of the division septum probably via its role in sPG wedge formation.

### The alpha isoform of PBP1b is enriched at the division site

Our results thus far suggest that PBP1b plays a specialized role in cell division distinct from its general function in PG biogenesis. Such a role would suggest that the enzyme is recruited to the division site to participate in the process. However, PBP1b has not been found to be strongly enriched at the division site. Immunofluorescence microscopy has previously observed a weak enrichment of PBP1b at midcell relative to the periphery in normally growing cells^22^. The enrichment is more pronounced in cells treated with the beta-lactam aztreonam^25^, but whether this localization reflects a division activity or a more general repair activity in response to PG damage caused by the division-specific PG synthesis inhibitor is not known. Furthermore, unlike the immunofluorescence results, green fluorescent protein (GFP) fusions to PBP1b displayed a peripheral localization pattern without a discernible enrichment at the division site^10^. Thus, whether PBP1b is specifically recruited to the septum to function in division has remained unclear.

**Fig. 4:**
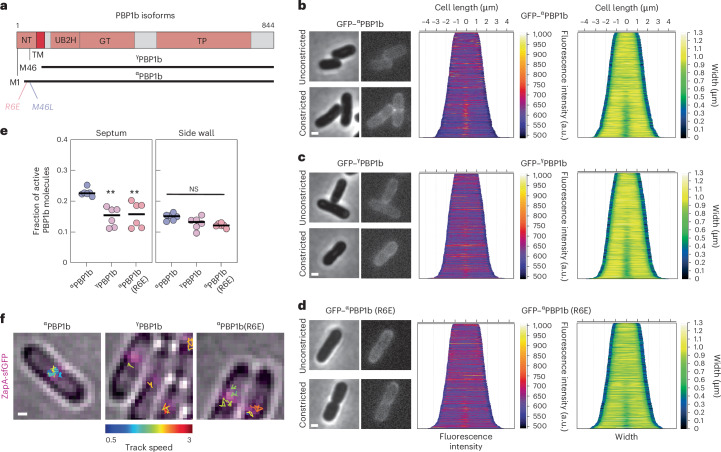
The longer alpha isoform of PBP1b is recruited to the division site. **a**, Primary structure, domain architecture and isoforms of PBP1b. NT, cytoplasmic N terminus; TM, transmembrane helix; UB2H, LpoB binding domain; GT, PG polymerase domain; TP, transpeptidase domain. **b**–**d**, Left: representative images of cells with or without an observable septal invagination. Demographs and snapshots are representative of 3 biological replicates. Middle: demographs (*N* = 395 (GFP–^α^PBP1b), 401 (GFP–^ɣ^PBP1b), 629 (GFP–^α^PBP1b(R6E)) showing localization of the indicated GFP–PBP1b isoform across the cell population arranged by increasing cell length (cell age). *X* axis shows cell length from midcell (0) to poles. Right: demographs of cell width measurements across their length. **e**, Mean of active fraction (stationary particles) of indicated PBP1b molecules as determined from SPT of Halo–PBP1b fusions acquired at 20 Hz. Significance was determined using one-way ANOVA with Tukey’s post test; septum: ^α^PBP1b vs ^ɣ^PBP1b **P = 0.0012, ^α^PBP1b vs ^α^PBP1b(R6E) **P = 0.0024, ^ɣ^PBP1b vs ^α^PBP1b(R6E) ^NS^*P* = 0.9303; side wall: ^α^PBP1b vs ^ɣ^PBP1b ^NS^*P* = 0.0619, ^α^PBP1b vs ^α^PBP1b(R6E) ^NS^*P* = 0.0513, ^ɣ^PBP1b vs ^α^PBP1b(R6E) ^NS^*P* = 0.6627. *N* = 6 SPT experiments from 2 biological replicates. **f**, Representative images of SPT tracking data for Halo–PBPB1b isoforms. Septal PBP1b SPT trajectories are overlayed onto a composite of 2 × 2 binned bright-field and GFP reference images. ZapA–GFP (false-coloured in magenta) signal served as a marker for division site localization. Scale bars, 0.5 µm. Source data

**Extended Data Fig. 5:**
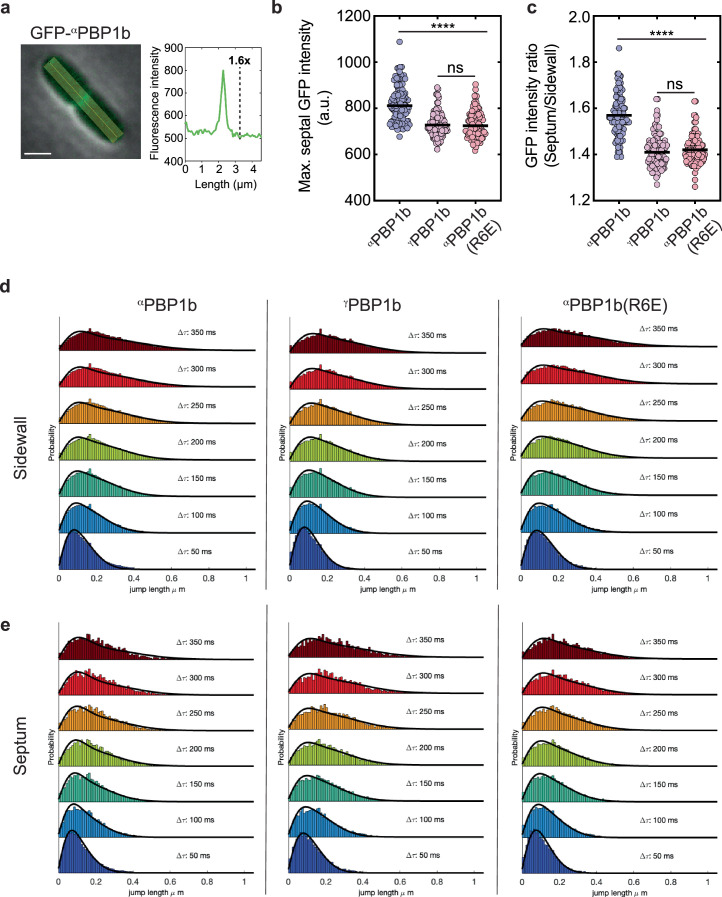
Preferential localization and activity of different PBP1b fusions. (**a**) Representative image of a cell expressing GFP-^α^PBP1b (AV266) induced with 50 µM IPTG. In constricting cells, GFP intensity was measured as a function of cell length and averaged over 10 pixels (0.65 µm). Scale bar = 1 µm. (**b**) Maximal septal GFP intensity, as well as the (**c**) ratio between the maximal (at the septum) and minimal (along the sidewall) intensity were plotted. Line represents median and significance was tested using one-way ANOVA with Tuckey’s posttest. Stars indicate significant differences, ns = non-significant. ^α^PBP1b vs ^γ^PBP1b: p = 1.044 × 10^−12^, ^α^PBP1b vs ^α^PBP1b(R6E): p = 1.041 × 10^−12^, ^γ^PBP1b vs ^α^PBP1b(R6E): p = 0.9533; ^α^PBP1b (N = 109), ^γ^PBP1b (N = 100), ^α^PBP1b(R6E) (N = 105). For the comparison of septal to side wall GFP intensities Kurskal-Wallis with Dunn’s posttest was applied. **** p < 1 × 10^−15^, ns = p > 0.9999. (**d,e**) Quantification of the stationary and diffusive fraction of single particle trajectories. Observed and fit distributions of particle jump lengths at the (**d**) side wall or (**e**) septum over eight steps with a 20 Hz acquisition frame rate (Δt = 50 ms) each, obtained using the Spot-On tool^42^. Source data

We wondered whether the difficulties defining the localization of PBP1b and its importance for cell division might be related to the observation that *E. coli* cells produce two isoforms of the enzyme^37–39^. The alpha form of PBP1b (^α^PBP1b) has a longer cytoplasmic N-terminal domain than the gamma form (^ɣ^PBP1b) for which translation is initiated 46 codons downstream of the ^α^PBP1b start (Fig. 4a). To determine whether the two isoforms have differential subcellular localization patterns, cells producing monomeric superfolder GFP (msfGFP) fusions to either ^α^PBP1b or ^ɣ^PBP1b were imaged. When either fusion was produced at low levels of induction (50 µM IPTG), newly born cells without an observable mid-cell constriction displayed a weak peripheral fluorescence signal (Fig. 4b,c). In longer cells with a constriction, the msfGFP–^α^PBP1b fusion appeared to be enriched at the division site relative to the peripheral membrane (Fig. 4b,c and Extended Data Fig. 5a–c). By contrast, the shorter msfGFP–^ɣ^PBP1b fusion maintained a largely peripheral localization pattern regardless of cell cycle stage (Fig. 4b,c and Extended Data Fig. 5a–c).

Previous analyses of aPBP dynamics detected two distinct populations of molecules, diffusive and stationary, with the stationary (bound) molecules being associated with PG synthesis function^14,15^. We therefore compared the single molecule trajectories of the two PBP1b isoforms using total internal reflection microscopy. Obtained tracks were fitted to a two-state kinetic model to determine the fraction of bound molecules^41–43^. To analyse the displacement of single PBP1b molecules at or in close vicinity to the Z-ring, ZapA–sfGFP was used as a fiducial marker. A similar fraction of active (that is, stationary/bound) molecules were detected along the side wall for both the Halo–^α^PBP1b and Halo–^ɣ^PBP1b fusions used for the tracking analysis (Fig. 4e, Extended Data Fig. 5a and Supplementary Video 6). However, when molecules in the region of the division site were analysed, a higher proportion of the Halo–^α^PBP1b molecules were active than those of Halo–^ɣ^PBP1b (Fig. 4e,f, Extended Data Fig. 5b and Supplementary Video 6). Thus, the longer N-terminal domain of the ^α^PBP1b isoform is associated with better mid-cell recruitment and higher activity at the division site.

### A predicted FtsA interaction recruits ^α^PBP1b to midcell

**Fig. 5:**
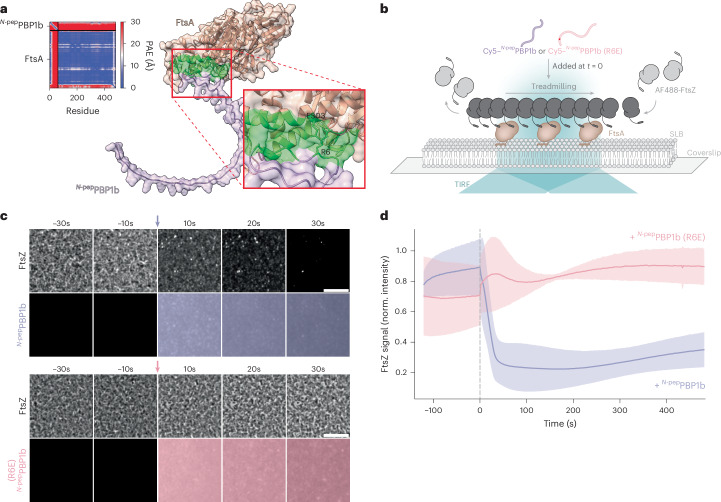
Evidence that the N-terminal domain of ^α^PBP1b interacts with FtsA. **a**, Right: predicted structure of FtsA (salmon) with ^*N*-pep^PBP1b (purple), with the salt bridge (^α^PBP1b(R6)–FtsA(E303)) at the interaction interface highlighted in green. Red box indicates the magnified region showing the interaction between FtsA–E303 and ^α^PBP1b–R6 (green). Left: predicted alignment error in Å of all residues against all residues for the top-ranked model (right). Low error (blue) corresponds to well-defined relative domain positions. **b**, Sideview schematic portraying experimental setup and protein components for experiments in **c** and **d**. **c**, Representative micrographs of AF488–FtsZ (grey), Cy5–^*N*-^^α^PBP1b (purple) and Cy5–^*N*-^^α^PBP1b (R6E) (pink). Arrows (*t* = 0 s) correspond to the time of addition of corresponding peptide. Scale bar, 5 µm. **d**, Mean intensity ± 1s.d. (shading) over time projections of normalized FtsZ fluorescence signal at the membrane before and after addition (*t* = 0 s) of Cy5–^*N*-pep^PBP1b (purple) and Cy5–^*N*-pep^PBP1b (R6E) (pink) plotted from 3 technical replicates.

**Extended Data Fig. 6:**
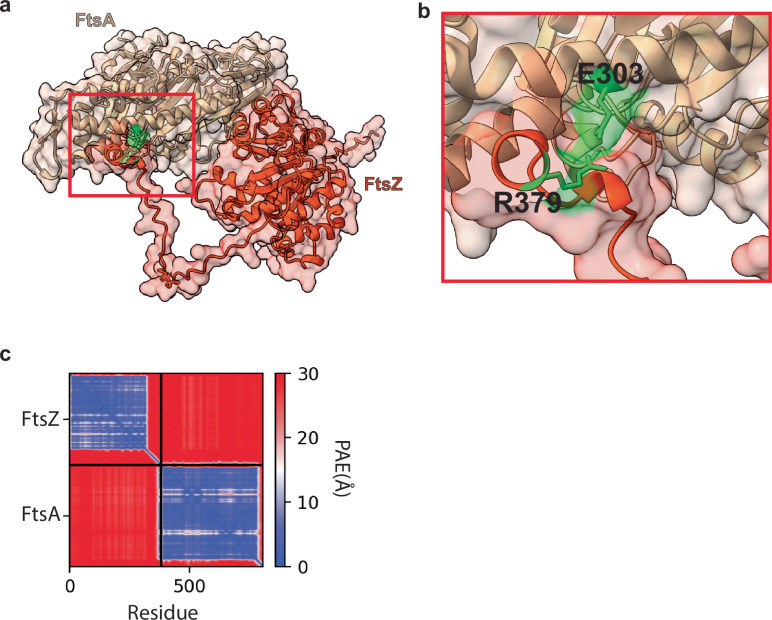
The FtsZ-FtsA interaction interface overlaps with the predicted ^N-pep^PBP1b-FtsA interface. (**a**) Predicted structure of an *E. coli* FtsA (salmon)-FtsZ (red) complex. Red box highlights the magnified region shown in (**b**). (**b**) Magnified image of (**a**) showing the interaction between FtsA E303 residue and FtsZ R379 residue (green). (**c**) Predicted alignment error in Å of all residues against all residues. Low error (blue) corresponds to well-defined relative domain positions. The binding position identified in the model mirrors that identified in the *Thermotoga* structure^88^.

To identify potential partners of ^α^PBP1b that promote its recruitment to the division site via interaction with its N-terminal cytoplasmic domain, we performed an in silico protein–protein interaction screen^44^. We were excited to see that the divisome protein FtsA was among the potential interaction partners of the N-terminal peptide of ^α^PBP1b but not of ^ɣ^PBP1b. The structural prediction suggests that the N-terminal peptide of ^α^PBP1b, referred to as ^*N*-pep^PBP1b, might bind FtsA with an interaction involving a salt bridge between R6 of ^α^PBP1b and E303 of FtsA (Fig. 5a). Notably, this region of FtsA is where the C-terminal peptide of FtsZ is bound to promote the association of FtsZ polymers with the inner face of the IM, suggesting that the N-terminal domain of PBP1b may compete with FtsZ for binding to FtsA (Extended Data Fig. 6).

**Extended Data Fig. 7:**
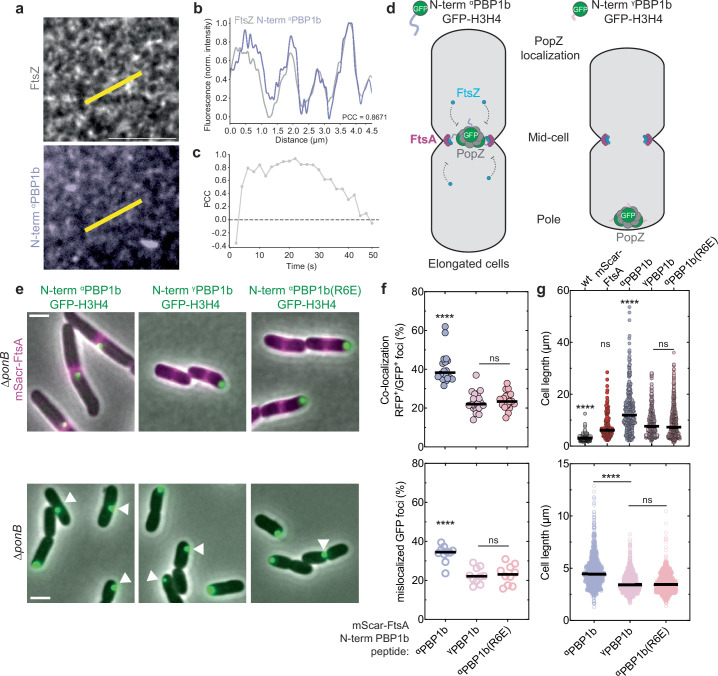
Transient colocalization of ^N-pep^PBP1b and FtsZ filaments in vitro and polar relocalization of N-term PBP1b constructs. (**a**) **Micrograph of a single frame shortly after addition of** ^**N-pep**^**PBP1b**. (**b**) **Fluorescent intensity profiles from AF488-FtsZ and Cy5-**^**N-pep**^**PBP1b corresponding to line ROI indicated in panel a. Pearson’s correlation coefficient (PCC) analysis was performed using numpy. corrcoef**^100^ **using the standard 2×2 correlation matrix of interpolated intensity profiles**. (**c**) PCC values over time between normalized intensities (maximum intensity normalization) of AF488-FtsZ and Cy5- ^N-pep^PBP1b in ROI from panel a, t = 0 s marks addition of Cy5- ^N-pep^PBP1b. (**d**) Schematic overview of localization of N-terminal PBP1b peptide fusions to GFP and the PopZ-interacting peptide H3H4 in cells producing an mScarlet-FtsA fusion and heterologously producing *Caulobacter crescentus* PopZ. This assay is based on the same principle as the POLAR two-hybrid assay^45^. (**e**) Top: Representative image of cells co-expressing PopZ with either ^N-pep^PBP1b (AV439) fused to GFP and H3H4 or similar fusions with the N-terminal peptide of ^γ^PBP1b (AV440) or ^N-pep^PBP1b(R6E) (AV441). The GFP fusions were produced from the P_ara_ promoter induced with 0.2 % arabinose. These cells also express mScar-FtsA under P_lac_ promoter control expressed from the *att*HK022 site induced with 250 µM IPTG. Bottom: cells expressing ^N-pep^PBP1b (AV442), ^γ^PBP1b (AV443) or ^N-pep^PBP1b(R6E) (AV444) in absence of mScar-FtsA. Arrowhead indicates mislocalized GFP foci. Scale bars = 2 µm. (**f**) Top: quantification of GFP signals overlapping with mScar-FtsA (RFP) at division sites. Significant differences relative to ^α^PBP1b were determined using one-way ANOVA with Dunnett’s posttest. Stars indicate significance, ns = non-significant. (^α^PBP1b vs ^γ^PBP1b: p = 6.238 × 10^−12^, ^α^PBP1b vs ^α^PBP1b(R6E): p = 3.0736 × 10^−11^, ^γ^PBP1b vs ^α^PBP1b(R6E): p = 0.9342). N = total number of images analyzed: ^α^PBP1b (N = 18), ^γ^PBP1b (N = 20), ^α^PBP1b(R6E) (N = 19). Bottom: quantification of mislocalized (non-polar) GFP foci; (^α^PBP1b vs ^γ^PBP1b: p = 2.0364 × 10^−5^, ^α^PBP1b vs ^α^PBP1b(R6E): p = 3.9258 × 10^−5^, ^γ^PBP1b vs ^α^PBP1b(R6E): p = 0.9671).^α^PBP1b (N = 10), ^γ^PBP1b (N = 10), ^α^PBP1b(R6E) (N = 10). (**g**) Top: quantification of cell length in indicated strains, wt and parental mScar-FtsA. Kurskal-Wallis with Dunn’s posttest was applied. Stars indicate significance, ns = non-significant. (WT vs mScar-FtsA: p < 1 × 10^−15^, WT vs ^α^PBP1b: p < 1 × 10^−15^, WT vs ^γ^PBP1b: p < 1 × 10^−15^, WT vs ^α^PBP1b(R6E): p < 1 × 10^−15^, mScar-FtsA vs ^α^PBP1b: p = 1.4491 × 10^−9^, mScar-FtsA vs ^γ^PBP1b: p > 0.9999, mScar-FtsA vs ^α^PBP1b(R6E): p > 0.9999, ^α^PBP1b vs. ^γ^PBP1b: p = 3.111 × 10^−11^, ^α^PBP1b vs. ^α^PBP1b(R6E): p = 5 × 10^−15^, ^γ^PBP1b vs. ^α^PBP1b(R6E): p > 0.9999). N = total number of cells analyzed: wt (N = 540), mScar-FtsA (N = 155), ^α^PBP1b (N = 326), ^γ^PBP1b (N = 442), ^α^PBP1b(R6E) (N = 555). Bottom: quantification of cell length in indicates strains, (^α^PBP1b vs. ^γ^PBP1b: p 1 × 10^−15^, ^α^PBP1b vs. ^α^PBP1b(R6E): p = 5 × 10^−15^, ^γ^PBP1b vs. ^α^PBP1b(R6E): p > 0.9999). ^α^PBP1b (N = 936), ^γ^PBP1b (N = 1126), ^α^PBP1b(R6E) (N = 976). Source data

To test for an FtsA interaction with ^*N*-pep^PBP1b, we took advantage of a previously developed assay for monitoring the recruitment of FtsZ protofilaments to supported lipid bilayers by FtsA polymers^5^ (Fig. 5b). Purified FtsA and fluorescent FtsZ were reconstituted on supported lipid bilayers and imaged. Importantly, FtsZ localization to the membrane in this assay is dependent on its interaction with FtsA^5^. Given that ^*N*-pep^PBP1b is predicted to compete with FtsZ for binding to FtsA, we used FtsZ membrane association as a proxy for the detection of a potential ^*N*-pep^PBP1b-FtsA interaction. Treadmilling FtsZ polymers were formed in the presence of FtsA on membrane bilayers doped with Ni-NTA-containing lipids. Upon the addition of His-tagged ^*N*-pep^PBP1b, the FtsZ polymers briefly co-localized with ^*N*-pep^PBP1b (Extended Data Fig. 7a–c) and then were rapidly dissociated from the membrane (Fig. 5c,d and Supplementary Video 7). However, FtsZ polymers at the membrane were largely unaffected by the addition of His-tagged ^*N*-pep^PBP1b(R6E) (Fig. 5c,d and Supplementary Video 8), which harbours a substitution predicted to disrupt the ^*N*-pep^PBP1b–FtsA interaction.

To further test the ^*N*-pep^PBP1b–FtsA interaction, we used the POLAR two-hybrid assay^45^. This cytological assay detects interactions via the ability of a bait protein associated with a polar PopZ focus to recruit a prey protein to the cell pole. An ^*N*-pep^PBP1b–GFP fusion was constructed with an H3H4 domain to target it to a polar focus of heterologously expressed PopZ. An mScarlet–FtsA fusion was co-expressed in these cells as the prey. Strikingly, rather than mScarlet–FtsA being recruited to a polar ^*N*-pep^PBP1b-GFP coated PopZ focus, the PopZ focus was frequently recruited to the mScarlet–FtsA labelled divisome (Extended Data Fig. 7d–f). In addition, cells expressing the ^*N*-pep^PBP1b–GFP–H3H4 fusion were elongated, indicating that the fusion impaired cell division (Extended Data Fig. 7g). Similar GFP–H3H4 fusions with ^*N*-pep^PBP1b(R6E) or the ^γ^PBP1b N-terminal peptide were not recruited to the division sites as frequently and did not cause a division defect beyond that caused by the production of mScarlet–FtsA alone (Extended Data Fig. 7d–g). Both the division phenotype and the mislocalization of ^*N*-pep^PBP1b–GFP–H3H4 to midcell were also observed in cells lacking the mScarlet–FtsA fusion (Extended Data Fig. 7d–g). Taken together, the biochemical and cytological assays support the AlphaFold2 (AF2) prediction that ^*N*-pep^PBP1b binds FtsA in its FtsZ-binding region and that the salt bridge between R6 of ^α^PBP1b and E303 of FtsA plays an important role in this interaction.

### Midcell ^α^PBP1b recruitment is critical for septal fortification

**Fig. 6:**
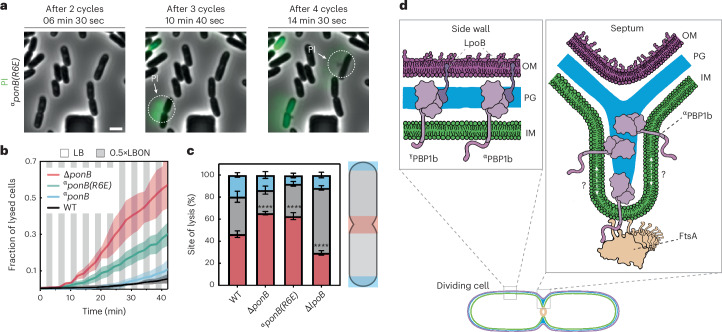
Septal localization of ^α^PBP1b is required for its function. **a**, Expression of the ^α^PBP1b(R6E) isoform was induced from a lactose promoter-controlled construct integrated at a phage attachment site in cells harbouring a deletion of the native *ponB* gene by the addition of 50 µM IPTG to growth media. Representative images of cell lysis events in response to osmotic oscillations between LB and 0.5×LB0N. Visualization of lysis was aided by imaging released DNA by propidium iodide (green) staining. Arrows point towards sites of septal lysis events. Scale bar, 2 µm. **b**, Mean fraction of lysed cells (line) ±1s.d. (shading) over time are plotted from *N* number of biological replicates: *N* = 3 (WT), 3 (*∆ponB*), 5 (^α^ponB(R6E)), 3 (^α^ponB). **c**, Manual quantification of subcellular lysis sites in response to osmotic shocks from 100 cells from 3 biological replicates. Data for *∆ponB* and *∆lpoB* are replotted from Fig. 3d and is represented as mean ± 1s.d. Significant differences relative to WT were determined using one-way ANOVA with Dunnett’s post test. WT vs ∆*ponB* ****P = 1.2094 × 10^−5^, WT vs ^α^ponB(R6E) ****P = 1.2085 × 10^−5^, WT vs *∆lpoB* ****P = 2.775 × 10^−5^. **d**, Summary schematic showing function of PBP1b during cell elongation and division, with the recruitment of ^α^PBP1b to the division site mediated by the predicted interaction with FtsA. It remains unclear where within the septum PBP1b functions (leading edge, lagging edge, or both), but it is presumably first recruited to the leading edge by FtsA. Fortification of the septum by PBP1b could take place there and/or it could be released from the leading edge to fortify regions of the septum at the lagging edge. It is also possible that a population of FtsA molecules associates with areas of the septum other than the leading edge to recruit PBP1b there as well. Source data

**Extended Data Fig. 8:**
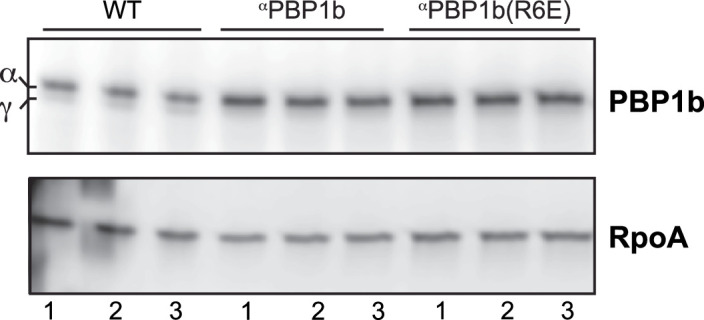
PBP1b isoforms are expressed at similar levels to WT. Protein levels were assessed by Western blot using polyclonal rabbit anti-PBP1b serum^10^ and anti-RpoA mouse monoclonal antibody Samples were from WT cells expressing native PBP1b and *∆ponB* cells expressing the indicated ^α^PBP1b variant at the same induction levels used for complementation experiments. RpoA served as a loading control. Molecular weight for ^α^PBP1b is 94.2 kDa (upper band) and for ^ɣ^PBP1b 88.9 kDa (lower band), respectively. The molecular weight of RpoA is 36.5 kDa. Protein sample from three biological replicated are displayed. Source data

To investigate the role of the predicted ^α^PBP1b–FtsA interaction on the recruitment of PBP1b to the division site, we compared the subcellular localization of GFP–^α^PBP1b(WT) and GFP–^α^PBP1b(R6E). Unlike the WT fusion, GFP–^α^PBP1b(R6E) was not strongly recruited to midcell (Fig. 4b–d). In addition, single-particle tracking (SPT) of the corresponding Halo fusions showed that a higher proportion of ^α^PBP1b(WT) was active at the division site relative to ^α^PBP1b(R6E) (Fig. 4e,f, Extended Data Fig. 5a,b and Supplementary Video 6). Moreover, cells expressing the ^α^PBP1b(R6E) showed the same increase in septal lesions in osmotic oscillation experiments as cells harbouring the full deletion of PBP1b (Fig. 6a–c, Extended Data Fig. 4d and Supplementary Video 5) despite its accumulation to similar levels as ^α^PBP1b(WT) (Extended Data Fig. 8). On the basis of these results, we conclude that the recruitment of ^α^PBP1b to the division site is required for its function and that this recruitment is likely to be mediated by the predicted ^*N*-pep^PBP1b–FtsA interaction.

### Conservation of the extended N-terminal peptide of ^α^PBP1b

**Extended Data Fig. 9:**
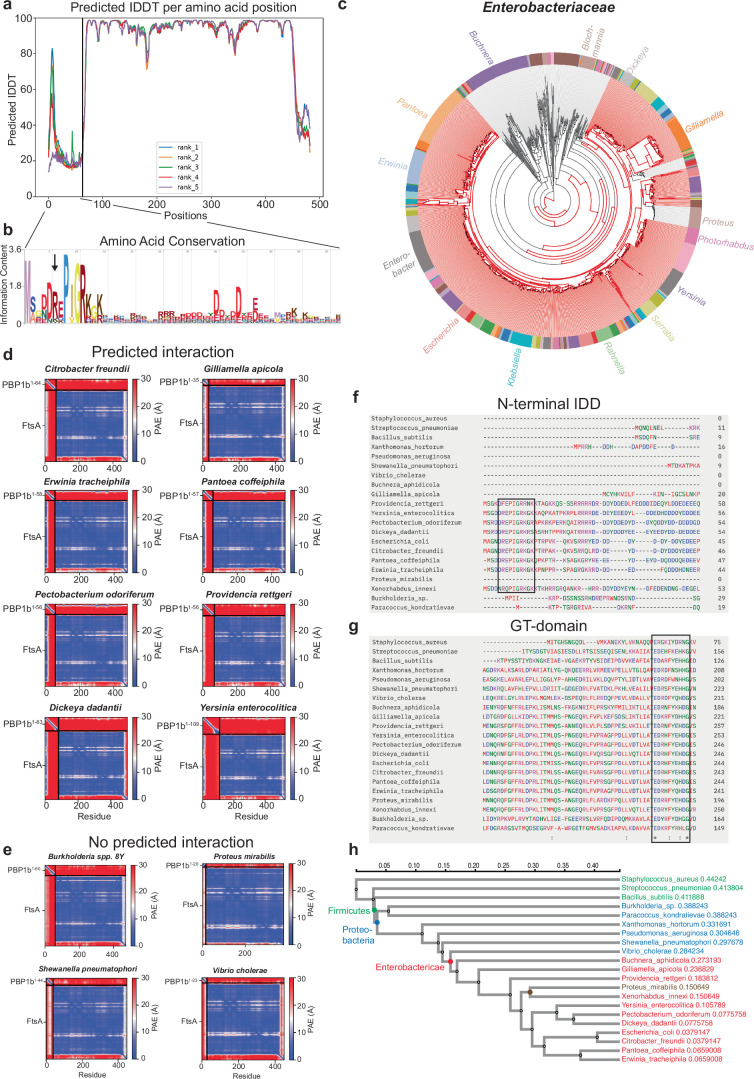
Conservation analysis of ^N-pep^PBP1b and its potential interaction with FtsA. (**a**) Predicted local distance difference test of ^N-pep^PBP1b and FtsA. (**b**) Amino acid conservation of ^N-pep^PBP1b sequence as identified by JackHMMER. Black arrow indicates R6. (**c**) Hits (in red) from JackHMMER^46^ search were visualized on a tree displaying the Enterobacteriaceae family using AnnoTree v1.2^93^. Closely related species that were not identified were added subsequently manually. (**d**) Additional examples of high-confidence predicted ^N-pep^PBP1b-FtsA complexes in distantly related Enterobacteriaceae. (**e**) AlphaFold predictions of ^N-pep^PBP1b-FtsA complexes using ^N-pep^PBP1b from Enterobacteriaceae family members not identified in the JackHMMER search as homologues (for example *Proteus mirabilis*) or PBP1b proteins found in other proteobacteria outside the Enterobacteriaceae family. Multiple sequence alignment (MSA) of full-length PBP1b proteins show amino acid conservation among distantly related bacteria for the (**f**) ^N-pep^PBP1b and the (**g**) catalytic GT domain. Black box highlights conserved amino acid sequence in a (sub)set of samples. (**h**) Phylogenetic tree reconstructed for MSA. Note the absence of ^N-pep^PBP1b sequence conservation in *P. mirabilis* (brown) (see also MSA in **f**).

**Extended Data Fig. 10:**
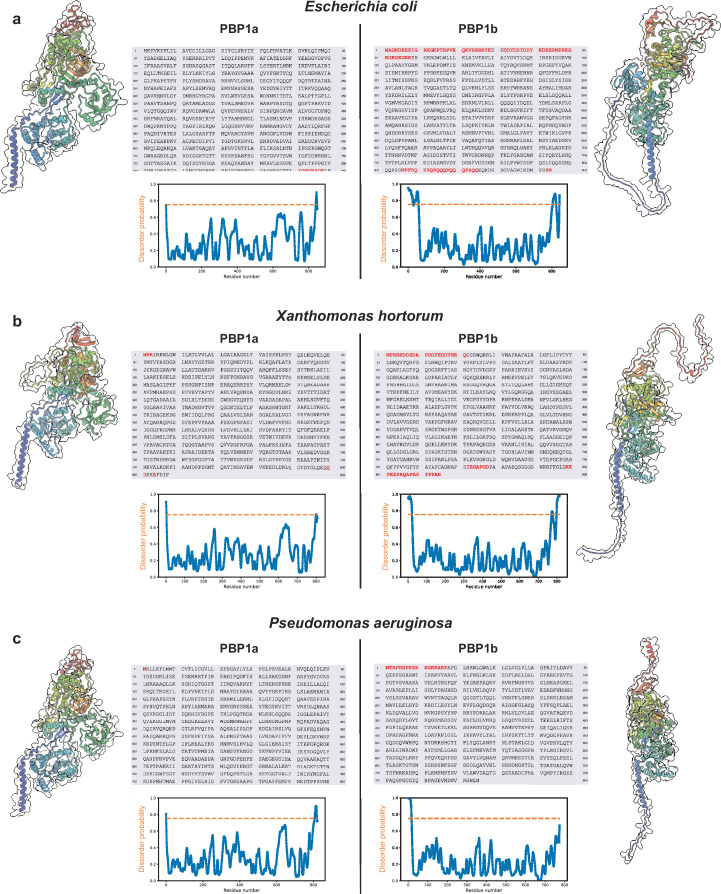
IDD domain prediction on aPBPs. AlphaFold and PrDOS highlights the presence of an on average 20-60 amino acid long N-terminal intrinsically disordered domain on PBP1b homologues which is absent on PBP1a homologues. PrDOS output displays predicted (<1% false positivity cutoff) disordered domains in red on the amino acid sequence. Protein structures were represented in ChimeraX and are rainbow colored depending on amino acid position from their N-terminus (blue) to C-terminus (blue). Representative images for (**a**) *E. coli* (Enterobacteriaceae), (**b**) *Xanthomonas hortorum* (Enterobacterales), and (**c**) *Pseudomonas aeruginosa* (Pseudomonata) are displayed.

To assess the conservation of the ^*N*-pep^PBP1b amino acid sequence, we searched for homologues using JackHMMER^46^ and obtained 113 hits clustered within the Enterobacteriaceae family (Extended Data Fig. 9a–c). By contrast, a similar analysis using the sequence of the glycosyl transferase (GT) domain of PBP1b resulted in 22,750 hits distributed across the entire bacterial domain. AF2 predictions indicated that selected homologues of ^*N*-pep^PBP1b also have the potential to interact with FtsA through the same residues as the *E. coli* peptide (Extended Data Fig. 9d). Surprisingly, although the first 14 amino acids of these peptides are conserved between organisms, the arginine at position six was more variable than the neighbouring residues P8, I9, G10 and R11, suggesting that these residues also contribute to the predicted ^*N*-pep^PBP1b–FtsA interaction as predicted by AF2. Importantly, non-homologous peptides from within (for example, *Proteus* spp.) or outside (for example, *Burkholderia, Vibrio, Shewanella* spp.) of the Enterobacteriaceae were not predicted to interact with FtsA. Notably, PBP1a homologues do not possess an intrinsically disordered N-terminal domain similar to the PBP1b homologues we tested, suggesting that the presence of such a domain may be predictive of septal recruitment (Extended Data Fig. 10). On the basis of this sequence analysis, we conclude that division site recruitment and septum fortification is likely to be a conserved function for PBP1b among the Enterobacteriaceae, with alternative recruitment mechanisms potentially promoting a similar activity in more distantly related bacteria encoding PBP1b variants with unrelated sequences at their N termini.

## Discussion

The FtsW–FtsI complex is essential for sPG synthesis during division in most characterized bacteria^1^. By contrast, the importance of aPBPs for division varies between organisms, which has made it difficult to determine their role in the process. In Gram-positive bacteria (Firmicutes), aPBPs range from being completely dispensable for division^47–49^, to supporting sPG production along with FtsW–FtsI in others^50^, to being the essential septum synthase^8^. Similarly, in Gram-negative bacteria (Proteobacteria), interactions between *E. coli* PBP1b and divisome components^22,23^ suggest that aPBPs play important roles in the division process, but the phenotypic consequences of aPBP inactivation also varies between these organisms. There are no readily observable division defects in *E. coli* when PBP1a or PBP1b is inactivated whereas in *Acinetobacter baumannii*, deletion of the gene encoding PBP1a results in mild cell filamentation and problems with daughter cell separation^51,52^. Even in cases where a division phenotype has been observable in cells lacking an aPBP, it is difficult to disentangle it from the general problems in PG synthesis displayed by mutants with aPBP defects. For example, PBP1b inactivation in *E. coli* results in a dramatic reduction in overall PG synthesis^14,40^. Thus, a decrease in PG labelling detected at midcell^27^ or a genetic interaction between an aPBP and a division gene^28–30^ could either reflect a direct division problem or an indirect one caused by a decline in PG content throughout the cell.

Here we report the discovery of a division-specific function for *E. coli* PBP1b. In situ cryo-ET indicates that this aPBP is required to produce a wedge-like structure of PG at the developing septum that contributes to its stiffness as shown by AFM. Furthermore, loss of this structure is associated with osmotic sensitivity and cell lysis via a loss of envelope integrity at the division site. Importantly, this activity of PBP1b is independent of its activator LpoB. Mutants lacking LpoB display all the general problems with PG biogenesis caused by PBP1b inactivation^10–12,16–19^ but not the defects associated with the loss of sPG wedge formation described here. Thus, our results indicate that PBP1b plays a specialized role in division site fortification that is distinct from its LpoB-dependent function in reinforcing the PG matrix throughout the cell cylinder (Fig. 6d). What activates PBP1b at the division site in place of LpoB is not currently known, but attractive candidates include one or more of the many PBP1b interaction partners identified previously^22–26^.

PG synthesis at the division site is thought to occur in two stages, either of which might involve sPG wedge formation by PBP1b. The first stage, referred to as pre-septal PG synthesis or PBP3(FtsI)-independent PG synthesis (PIPS), involves the localized insertion of PG at midcell to promote zonal cell elongation^53–57^. This step is followed by the FtsW–FtsI-dependent transition to the inward growth of sPG and the accompanying invagination of the membrane. Pre-septal PG synthesis occurs independently of many cell division proteins, including FtsI and FtsA, but depends critically on FtsZ and Z-ring formation^55,56^. The process also occurs in cells individually inactivated for either PBP1a or PBP1b^56^. Thus, if aPBPs indeed play a critical role in pre-septal PG synthesis as some results suggest^25^, the two different enzymes are interchangeable for this activity. By contrast, sPG wedge formation and division site stabilization were found to be a PBP1b-specific function. In addition, our results suggest that PBP1b is recruited to the division site to promote sPG wedge formation via an interaction with FtsA, which is not required for pre-septal PG synthesis^56^, and the wedge-like sPG structure was only observed in cryo-electron tomograms of cells with constricted membranes^31^. We therefore conclude that the division site stabilizing activity of PBP1b reported here is operating contemporaneously with sPG ingrowth promoted by FtsW–FtsI rather than pre-septal synthesis. How the activities of PBP1b and FtsW–FtsI are related to and/or coordinated with each other in space and time during this phase of division remains to be determined. However, an attractive possibility is that the mechanism resembles a previous proposal for Gram-positive bacteria^58^ in which FtsW–FtsI produces a core of sPG that is sandwiched by fortifying layers produced by PBP1b. Notably, it may be differences in the relative importance of the fortifying sPG layer that contribute to the variable severity of division phenotypes associated with aPBP inactivation in different bacteria.

Finally, our results demonstrated not only a division-specific role for PBP1b, but one that depends on a specific isoform of the enzyme (Fig. 6d). It has long been known that the *ponB* gene encoding PBP1b has two start codons that produce either the longer ^α^PBP1b or the shorter ^ɣ^PBP1b isoform^37–39^, but the functional relevance of the two forms has remained unclear. Here we show that it is the extended N terminus of the alpha isoform that is needed for the recruitment of PBP1b to midcell for division site fortification. Notably, a previous study found that the alpha isoform of PBP1b was specifically required to prevent cell lysis when PBP1a and FtsI(PBP3) were simultaneously targeted with beta-lactam antibiotics^39^. The authors also found that this activity involved the first six residues of ^α^PBP1b and astutely suggested that they may function to promote an interaction with the division apparatus^39^. Our results confirm this suggestion and argue that the recruitment of ^α^PBP1b to the divisome involves an interaction between ^*N*-pep^PBP1b and FtsA, with residue R6 of ^α^PBP1b making a critical contact with FtsA. Although this interaction requires further biochemical and structural validation, the observation that high-confidence interactions are also predicted for ^*N*-pep^PBP1b–FtsA pairs from other enterobacteria supports the recruitment model and suggests that the mechanism is likely to be conserved. Regardless of the exact interaction partner, the extended cytoplasmic N terminus of ^α^PBP1b is clearly required for the recruitment. Many aPBPs encoded by diverse bacteria have extended cytoplasmic N termini, and in Firmicutes these termini are also involved in division site localization via interactions with the Gram-positive division protein GpsB^59^. Therefore, although the details are likely to differ, the division site recruitment and fortification mechanism reported here for *E. coli* PBP1b may be widespread among bacteria.

## Methods

### Media, bacterial strains and mutagenesis

All strains used in this study are derivatives of *E. coli* MG1655. Strains and plasmids used in this study are listed in Supplementary Tables 2 and 3. Primers used for PCR are listed in Supplementary Table 4. Bacteria were grown in LB (1% tryptone, 0.5% yeast extract, 0.5% NaCl), 0.5×LB0N (0.5% tryptone, 0.25% yeast extract) or M9 medium^60^ supplemented with 0.2% D-glucose and casamino acids each. For selection, antibiotics were used at 10 µg ml^−1^ (tetracycline), 25 µg ml^−1^ (chloramphenicol) and 50 µg ml^−1^ (kanamycin, ampicillin). Mutant alleles were moved between strains using phage P1 transduction or integrated by expression of the integrase from temperature-sensitive plasmid (pTB102)^61^. If necessary, the antibiotic cassette was removed using FLP recombinase expressed from pCP20 (ref. ^62^). All mutagenesis procedures were confirmed by PCR and Sanger sequencing.

### Cryo-EM specimen preparation

Bacterial strains were grown overnight in LB media, back diluted 1:1,000 and grown with shaking at 37 °C and 250 r.p.m. to an optical density at 600 nm (OD_600_) = 0.3. Cells were collected by centrifugation (2 min, 5,000 × *g*, r.t.) and resuspended in LB media to a final OD_600_ = 0.6. This cell suspension (3 µl) was applied to Cflat-2/1 200 mesh copper or gold grids (Electron Microscopy Sciences) that were glow discharged for 30 s at 15 mA. Grids were plunge frozen in liquid ethane^63^ with an FEI Vitrobot Mark IV (Thermo Fisher) at r.t., 100% humidity, with a waiting time of 13 s, one-side blotting time of 13 s and blotting force of 10. Customized parafilm sheets were used for one-side blotting. All subsequent grid handling and transfers were performed in liquid nitrogen. Grids were clipped onto cryo-FIB autogrids (Thermo Fisher).

### Cryo-FIB milling

Grids were loaded in an Aquilos 2 Cryo-FIB-SEM microscope (Thermo Fisher). The specimen was sputter coated inside the cryo-FIB chamber with inorganic platinum, and an integrated gas injection system (GIS) was used to deposit an organometallic platinum layer to protect the specimen surface and avoid uneven thinning of cells. Cryo-FIB milling was performed on the specimen using two rectangular patterns to mill top and bottom parts of cells^64,65^, and two extra rectangular patterns were used to create micro-expansion joints to improve lamellae stability^66^. Cryo-FIB milling was performed at a nominal tilt angle of 14°–18° which translates into a milling angle of 7°–11°. Cryo-FIB milling was performed in several steps of decreasing ion beam currents ranging from 0.5 nA to 10 pA and decreasing thickness to obtain 150–250 nm lamellae.

### Cryo-electron tomography

All imaging was done on an FEI Titan Krios (Thermo Fisher) transmission electron microscope operated at 300 KeV equipped with a Gatan BioQuantum K3 energy filter (20 eV zero-loss filtering) and a Gatan K3 direct electron detector. Before data acquisition, a full K3 gain reference was acquired, and ZLP and BioQuantum energy filter were finely tuned. The nominal magnification for data collection was ×33,000, giving a calibrated 4 K pixel size of 2.758/2.704 Å. Data collection was performed in the nanoprobe mode using the SerialEM or Thermo Scientific Tomography 6 software. The tilt range varied depending on the lamella, but was generally from −68° to 68° in 2° steps following the dose-symmetric tilt scheme. Tilt images were acquired as 8 K × 11 K super-resolution movies of 4–8 frames with a set dose rate of 1.5–3 e^−^ Å^−1^ s^−1^. Tilt series were collected at a range of nominal defoci between −3.5 and −5.0 µm and a target total dose of 80–180 e^−^ Å^−2^ (Supplementary Table 1).

### Cryo-electron tomography image processing

Acquired tilted super-resolution movies were motion corrected and Fourier cropped to 4 K × 5 K stacks, using framealign in IMOD^67^. Tilt series were aligned using etomo in IMOD^68^ and Dynamo^69^. Contrast transfer function (CTF) estimation was performed in IMOD^70^. CTF correction was performed with the ctfphaseflip programme in IMOD. CTF-corrected unbinned tomograms were reconstructed by weighted back projection and subsequently 2×, 4× and 8× binned in IMOD. Denoising of cryo-ET data was performed in cryo-CARE^71^. Bandpass filtering and summed projection of cryo-tomogram slices was performed in Dynamo complemented with customized MATLAB scripts^72^.

#### Segmentation

Segmentation was performed on binned 4× denoised tomograms using Amira (Thermo Fisher) for the cell wall signal by non-biased semi-automatic approaches. Manual annotation was required every 10 slices, then Amira’s interpolation function was applied to automatically trace slices in between. Annotation was done in 2D slices where features of interest were visible by eye. The segmented PG signal is not indicative of a specific glycan strand network but rather serves as a visual guide to relevant densities assigned to the cell wall. IM and OM were segmented using MemBrain^73,74^.

#### Template matching

Dynamo’s template matching module was used to pick ribosomes in binned 4× cryo-electron tomograms and assigned angles. The ribosome template was adjusted to the pixel size of the tomogram in Dynamo^72,75^. Rendering of Dynamo tables from the template matching process was done in ArtiaX^76^, a plugin of ChimeraX^77^.

### Quantification of cryo-ET data

Summed projection images of binned 4× cryo-ET tomograms were used to quantitatively measure cell dimensions at the division site^78^.

#### Periplasmic space

Measurements of periplasmic space thickness (septal width) were performed from centre to centre of opposing IM at the septum^31^ (commonly, both sides of the division sites were measured, yielding two values per septum). We used a customized macro in FIJI^79^ that measures 30 Euclidean distances from surface-to-surface areas^80^, for example, from IM to IM at the septum (in nm) and spaced evenly along the IM signal line. For these 30 single measurements, the mean and standard deviation were calculated, yielding a final single value per septum.

#### IM–OM distance

Measurements were performed in FIJI using the ‘point to point’ measuring tool. Measurements were done from IM tip to OM tip (IM–OM distance) at the septum in FIJI^79^ (both sides of the division site were measured).

#### Tangent to OM

Measurements were performed in FIJI using the ‘point to point’ measuring tool. The shortest tangent line to the OM was drawn from IM to IM at the envelope invaginations. The line was drawn on both sides of the division site, yielding two tangent lines per division site. The difference in length between these lines was used to report division site symmetry. Commonly, two sides of the division site are visible in the cryo-electron tomograms, thus, one cryo-electron tomogram yielded two measurements unless one side of the division site was not visible due to imaging limitations (for example, ice deposition or shifting during acquisition).

### 3D rendering

3D rendering and movies were performed in ChimeraX and ArtiaX^76,77^.

### Isolation of PG sacculi for AFM

Overnight cultures of the indicated strains were back diluted 1:1,000 into 50 ml of fresh LB and grown at 37 °C until OD_600_ = 0.3–0.4. Cells were collected by centrifugation (2 min, 5,000 *g*) and fixed in ice-cold 70% ethanol for 20 min. After three washes with double-distilled water (ddH_2_O), cells were boiled with stirring in 4% SDS for 45 min. Sacculi were further washed (3× ddH_2_O) and treated with 200 µg ml^−1^ Pronase E (Sigma Aldrich, P5147) in 10 µM Tris-pH 7.8 containing 0.5% SDS for 2 h at 60 °C. Finally, sacculi were washed 3× in ddH_2_O and stored in 500 µl of ddH_2_O containing 0.02% sodium azide.

### AFM

For AFM analysis, microscope slides were pretreated as previously described^81,82^. Briefly, slides were plasma cleaned (Harrick Plasma PDC-32G-2; 2 min, high setting) and coated with Vectabond (Vector Laboratories, SP-1800-7) according to manufacturer protocol. Approximately 50 µl of 1:10 diluted (in ddH_2_O) sacculi were added onto a Vectabond-coated slide and allowed to adhere for 5 min before rinsing 3× with ddH_2_O.

AFM was performed at the Harvard Center for Nanoscale Systems using a JPK Nanowizard IV with UltraSpeed head (Burker). Mechanical measurements were performed in QI mode with a qp-BioAC CB3 cantilever (NanoAndMore, qp-BioAC-10) with a nominal spring constant of 0.06 N m^−1^ and 30 kHz resonant frequency. The cantilever stiffness was calibrated by measuring the thermal noise of the cantilever in ddH_2_O. First, low-resolution 10 µm overview scans of sacculi were taken using 256 × 256 pixels. For high-resolution imaging, 1 μm^2^ scans of sacculi were taken with 128 × 128 pixels, 0.2 nN set point, 1,000 nm *z*-length and 62.5 µm s^−1^ *z*-speed. Sacculi displaying a distinguishable septal band and absence of PG folding were imaged at high resolution (1 µm, 128 × 128 pixels). The ‘height’ measurement reflects the sample topography as inferred from how much the Z-piezo moves up and down relative to the setpoint (0.2 nN).

For mechanical measurements, images were analysed in the JPK data-processing software. The effective Young’s modulus was calculated using the Hertz–Sneddon model assuming a pyramidal tip shape, a radius of 2 nm and a Poisson ratio of 0.5. Stiffness of the division site was quantified in FIJI^79^ by detecting septal bands on low-resolution height images using default thresholding function. Two 300 × 200 nm regions of interest were manually added adjacent to the septal band (Fig. 2b,c). Average Young’s modulus for the stiffness of the PG was determined by taking the ratio from the septal band and the side wall (Fig. 2b). Absolute Young’s moduli for PG stiffness at the indicated regions (septal band versus side wall) are displayed in Extended Data Fig. 3b. PG pore size was determined from high-resolution height images in FIJI^79^ (Fig. 2d). First images were processed with a 2D difference of Gaussian filter (sigma 1 = 2 pixel; sigma 2 = 3 pixel) and subsequently imported as a mask into MicrobeJ^83^ for detecting pores using thresholding and interpolation functions. All images were rendered for publication using JPK data-processing software.

Please note that the mechanical properties of the material and sample height are two independent features. Stiffness reflects the local mechanical response of the material (for example, composition, crosslinking, or underlying support), which is not influenced by elevated topography (for example, folding of PG at the division site)

### Bacterial growth assays

To assess the effect of PBP1b inactivation on viability, cells were osmotically challenged by growth in 0.5×LB0N at 42 °C. Briefly, indicated strains were grown overnight at 30 °C in LB supplemented with tet10 and 250 µM IPTG when necessary. The next morning, cells were back diluted 1:1,000 in fresh LB at 37 °C and grown until OD_600_ = 0.3–0.4 in the presence of inducer when necessary. Cells were collected by centrifugation (2 min, 5,000 *g*) and normalized to OD_600_ = 1.

For spot titres, cells were plated in 10-fold dilutions on LB or 0.5×LB0N plates and incubated at 42 °C for 18 h. To assess the minimal levels of ectopic PBP1b production needed for complementation, cells harbouring the relevant P_lac_ promoter driven expression were plated on 0.5×LB0N agar containing 0.2% glucose, 50 µM, 100 µM or 250 µM IPTG, and incubated at 42 °C (data not shown). Complementation was observed at 50 µM IPTG for the WT PBP1b expression construct (Extended Data Fig. 4b). Growth curves in liquid medium were measured in a Tecan M-plex (Tecan) 96-well plate reader by back diluting day cultures to OD_600_ = 0.01 in LB or 0.5×LB0N. Multiwell plates were incubated with shaking at 42 °C for a total of 6 h.

### Microfluidic osmotic shock assay

Osmotic oscillations were performed to assess the mechanical integrity of the cell envelope as previously described^84,85^. Overnight cultures of the indicated strains were back diluted 1:500 into fresh LB and grown until OD_600_ = 0.3–0.4 at 37 °C. Subsequently, cells were diluted to OD_600_ = 0.1 and loaded into a CellASIC bacterial microfluidic perfusion plate (Merck, B04A) according to manufacturer protocol. The plate was then transferred to a Nikon Ti2E inverted microscope equipped with a preheated (37 °C) Oko-lab environmental enclosure and allowed to acclimate for at least 30 min. Cells were exposed to osmotic shocks by oscillating the growth medium from LB to 0.5×LB0N ten times during a 42-min observation window with a 10-s acquisition frame rate using the ONIX flow control (Merck). This resulted in a total of 10 hypo-osmotic (LB to 0.5×LB0N) and hyper-osmotic (0.5×LB0N to LB) shocks. Cell envelope integrity was monitored by adding 5 µM SYTOX-Green nucleic acid stain (Thermo Fisher) or propidium iodide to the growth media. Phase-contrast and fluorescence images were recorded using a Plan Apo ×100/1.45 Oil Ph3 objective lens, a Lumencor Spectra III Light Engine illumination and GFP/RFP specific filter set (Semrock quad-band dichroic LED-DA/FI/TR/Cy5/Cy7-5X-A-000 and Semrock FF01-515/30 or Semrock FF01-641/75 emission filters), a Hamamatsu ORCA-Flash4.0 V3 sCMOS camera and Nikon Elements 5.2 acquisition software. The resulting time-lapse movies were drift corrected using a customized StackReg plugin in FIJI^86,87^. Cell lysis events were quantified by counting the number of newly appearing SYTOX signals every 10th frame using the ‘Find Maxima’ function in FIJI. Sites of cell lysis events were determined from phase-contrast and SYTOX-Green images by three independent laboratory members.

### Generation of PBP1b variants

CRIM vectors^61^ for expressing WT PBP1b (pAV12) and the GT catalytic point mutant (pAV16) expression constructs were generated as follows: *ponB* was amplified from genomic DNA using primers ponB_XbaI-Fw and ponB_BmtI-rev (Supplementary Table 4). The resulting PCR product and pHC949 were digested using XbaI and BmtI and ligated to generate pAV12. The catalytic mutant (ponBE233D) was generated using KLD (NEB) according to manufacturer instructions with primers KLD_ponBE233D_Fw and KDL_ponBE233D_rev to generate pAV16.

CRIM vectors expressing N-terminal GFP/Halo fusions to ^ɣ^PBP1b from the P_lac_ promoter were previously generated (pHC942 and pHC949)^14^. In addition, full-length *ponB* was amplified from genomic *E. coli* DNA using primers (ponB-alpha-BamHI and ponB-NheI_rev) (Supplementary Table 4) and cloned into pHC942 and pHC949 using BamHI (NEB, R3136T) and NheI (R3131S). To express the stable ^α^PBP1b isoform, the second start codon was mutated to a leucine (M46L) using primers ponB-M46L_FW and ponB-M46L_rev (Supplementary Table 4) with the Q5 Site-Directed Mutagenesis kit (NEB, E0552S). Ultimately, this procedure resulted in the plasmid pAV13 (Halo–PBP1b-alpha) and pAV15 (msfGFP–PBP1b-alpha). Similarly, the R6E point mutation was introduced for the generation of pAV35 (msfGFP–PBP1b-alpha R6E) and pLM001 (Halo–PBP1b-alpha R6E) using primer pairs ponB-alpha-R6E-pAV15_rev and ponB-alpha-R6E-pLM01_rev, respectively. For the expression of untagged PBP1b isoforms and R6E point mutants, pAV13 was digested with XbaI/PstI and re-ligated with the PCR product of ^α^ponB amplified from its backbone using primers XbaI_RBS-ponB-alpha-FW and ponB_rev_PstI (Supplementary Table 4). CRIM vectors were transformed into the *∆ponB* background (TU122) and integrated into the chromosome at the *att*HK022 site using the temperature-sensitive helper plasmid pTB102 (ref. ^61^). Single integration events were confirmed by colony PCR and the helper plasmid was cured by incubation at 37 °C.

### Assessing PBP1b levels by immunoblotting

To assess the expression levels of different PBP1b isoforms and point mutants, overnight cultures of the indicated strains were back diluted 1:1,000 into LB and grown to OD_600_ = 0.3–0.4 at 37 °C. Cells were collected by centrifugation (2 min, 5,000 *g*), concentrated to 3 OD_600_ units ml^−1^ and resuspended in 50 µl PBS containing EDTA-free Protease Inhibitor (Roche, 11836170001) and mixed 1:1 with 2× Laemmli Sample Buffer (Bio-Rad, 1610737). Samples were sonicated and denaturated at 100 °C for 10 min. Total protein concentration for each sample was determined using the non-interfering protein assay (G Biosciences, 786-005) according to instructions. Total protein (5 µg ml^−1^ per lane) were loaded onto 4–20% Mini- PROTEAN SDS-PAGE gel (Bio-Rad, 4561094) and run at 150 V for 1 h. The gel was transferred to a PVDF low-fluorescence membrane using the Trans-Blot Turbo Transfer System (Bio-Rad) and blocked with 5% milk in PBS containing 0.05% Tween20 (M-PBS-T). Proteins were detected with primary antibodies (polyclonal rabbit α-PBP1b serum diluted 1:10,000 (ref. ^10^) or monoclonal mouse α-RpoA antibody diluted 1:10,000) (BioLegend, 663104) and incubated for 1 h in 1% M-PBS-T. The membrane was then washed 3 times with 0.05% PBS-T, incubated with secondary antibody solution (IRDye 800CW anti-rabbit IgG goat secondary antibody and IRDye 680RD goat anti-mouse IgG secondary antibody, LI-COR Biosciences) in 0.5% M-PBS-T for 1 h at room temperature and washed again 3 times with 0.05% PBS-T. The blot was visualized using a fluorescence imager (Bio-Rad, ChemiDoc MP).

### Wide-field fluorescence microscopy

To visualize the localization of PBP1b isoforms, indicated strains were grown overnight in LB-tet10 at 30 °C. Cells were washed 3×, back diluted 1:1,000 in M9-Glu-CAA supplemented with 50 µM IPTG and grown until OD_600_ = 0.3–0.4 at 37 °C. Samples were collected by centrifugation (2 min, 5,000 *g*) and immobilized on a 2% agarose in an M9-Glu-CAA pad and covered with a coverslip. Cells were imaged on a Nikon Ti-E inverted wide-field microscope equipped with a fully motorized stage and perfect focus system and custom-made environmental enclosure heated to 37 °C. Images were acquired using a 1.45 NA Plan Apo ×100 Ph3 DM objective lens with Cargille Type 37 immersion oil. Fluorescence was excited using a Lumencore SpectraX LED light engine (50 ms exposure, 100% LED power) and filtered using the ET-GFP (Chroma, 49002) filter set. Images were recorded on an Andor Zyla 4.2 Plus sCMOS camera (65 nm pixel size) using Nikon Elements (v.5.10) acquisition software. Images were analysed and rendered for figure or movie display using FIJI^79^ and demographs were generated using MicrobeJ (v.5.13 d)^83^. In 100 randomly chosen constricting cells (determined by phase contrast), fluorescence intensities across cell length for distinct GFP–PBP1b constructs were measured manually (Extended Data Fig. 5a). From these intensity traces, maximal (Extended Data Fig. 5b) and minimal fluorescence were extracted, and their ratio was taken as corresponding to the relative septal enrichment of the protein fusion (Extended Data Fig. 5c). Original images and detection parameters were uploaded on the Zenodo repository (link will be accessible upon publication).

### Total internal reflection microscopy for SPT of PBP1b isoforms

Overnight cultures of indicated strains were back diluted 1:1,000 in LB supplemented with Tet10 and Cam25, and grown at 37 °C until OD_600_ = 0.2–0.3. Janelia Fluor Halo-ligand JF549 (Promega, GA1110) was added at 20 nM final concentration, while incubation of cells was continued for an additional 20 min. Subsequently, 1 ml of cells was collected by centrifugation (2 min, 5,000 *g*) and washed 3× in 1 ml LB before resuspending in 30 µl of LB. Cells were added on a plasma-cleaned (Harrick Plasma PDC-32G-2; 1 min, high setting) #1.5H coverslip (Marienfeld, 0107052) and immobilized on 2% (w/v) agarose in an LB pad.

TIRF microscopy was carried out on a Nikon Ti inverted microscope equipped with a fully motorized stage, 488 and 561 laser lines, Chroma ET GFP (49002) and mCherry (49008) filter cubes, ApoTRIF ×100 1.49NA objective with correction collar, and a stage-top incubator (Oko-lab) heated to 37 °C. The microscope was controlled using the Nikon Elements (4.30) software and SPT movies were recorded on an Andro Zyla 4.2 Plus sCMOS camera (Oxford Instruments) using 2 × 2 binning with 50 ms exposure and 30% laser power for a total duration of 30 s, resulting in an effective acquisition frame rate of 20 Hz. Single bright-field and ZapA–GFP reference images were acquired after each movie.

### SPT analysis

Particle tracking was performed in FIJI^79^ using TrackMate (v.7.11.1)^41^. Foci were detected using the LoG detector with an estimated object diameter of 0.4 and an initial quality threshold of 10. Spurious spots outside cells were filtered using intensity-based thresholding of bright-field signal. Foci were further filtered using intensity-based thresholding of the ZapA–GFP signal to compare PBP1b dynamics at the septum (high GFP signal) or the side wall (low GFP signal). Foci were linked using the LAP Tracker with a maximum linking distance of 0.4 µm and a maximum frame gap of 2. Tracks consisting of at least 3 spots were exported and further analysed using the Spot-ON website^43^. To estimate the populations of immobile and freely diffusing molecules, tracks were fit to a two-state model. For all conditions, the fit parameters were left free to ensure unbiased estimates. Additional SPT statistics are summarized in Supplementary Table 6.

### AlphaFold multimer screen for PBP1b isoforms

To identify protein interaction partners for the cytoplasmic N termini of the different PBP1b isoforms, an AlphaFold multimer screen was performed. To this end, the amino acid sequences of the PBP1b alpha (1–60: MAGNDREPIGRKGKPTRPVKQKVSRRRYEDDDDYDDYDDYEDEEPMPRKGKGKGKGRKPR) and gamma (46-60: MPRKGKGKGKGRKPR) were run against the full *E. coli* K12 reference proteome (UniProt Proteome ID UP000000625). Each pair was folded with three of the five AlphaFold-Multimer models, resulting in three independent predictions for each protein pair. Hits were defined as pairs where any of the three models had any inter-chain residues with two non-hydrogen atoms positioned within 8 Å; a predicted local distance difference test (pLDDT) > 50; and a predicted alignment error (PAE) value of <15 Å. The results of the multimer screen are provided in Supplementary Data 1. This screen yielded 579 ‘hits’ out of 8,466 pairwise interactions (442 (alpha) vs 137 (gamma), see Supplementary Data 1). Hits were manually curated as many probably correspond to false positives given that they reside in the wrong cellular compartment (for example, periplasm and OM).

Interactions between ^*N*-pep^PBP1b and FtsA were further verified in selected species on the basis of sequence conservation analysis by JackHMMER (see below) using the publicly available ColabFold v.1.5.5 webserver (https://colab.research.google.com/github/sokrypton/ColabFold/blob/main/AlphaFold2.ipynb) with default parameters. The FtsZ–FtsA binding position (Extended Data Fig. 6) is based on the identified site in the structure from Thermotoga^88^.

### Polar expression of ^*N*-pep^PBP1b constructs

To assess the interaction between different N-terminal peptides of PBP1b and FtsA in vivo, we ordered synthetic DNA fragments encoding the sequence of peptides of α (1–63, M46L, pAV48), γ (46–63, pAV49) and αR6E (1–63, R6E, M46L, pAV50) fused to GFP–H3H4 as GeneBlocks (IDT) (Supplementary Table 5). The fragments were amplified using primers ^*N*-pep α^PBP1b-FW and ^*N*-pep α^PBP1b-rev, ^*N*-pep γ^PBP1b-FW and ^*N*-pep α^PBP1b-rev, and ^*N*-pep α^PBP1bR6E-FW and ^*N*-pep α^PBP1b-rev (Supplementary Table 4), and ligated into XbaI/Hind III digested pHCL149 (ref. ^45^) (Supplementary Table 3). The resulting vector encodes PopZ and a C-terminal GFP–H3H4 fusion under control of the arabinose promoter. Due to the strong interaction of the 52-amino-acid-long H3H4 homo-oligomerization domain and PopZ, one can recruit a protein of interest to the cell pole (Extended Data Fig. 7d)^89–91^.

The *ftsA* gene was obtained by digesting pHCL425 with BamHI/HindIII and cloned into similarly digested pHCL152. The resulting vector (pAV41, Supplementary Table 3) encodes an IPTG-inducible N-terminal fusion of mScarlet to FtsA which is integrated at the phage *att*HK022 attachment site.

The constructs were transformed in strains harbouring a clean deletion of *ponB* (AV250, Supplementary Table 2) to generate AV442–AV443 (Supplementary Table 2) as well as an inducible mScarlet fusion to FtsA (RA006, Supplementary Table 2) to generate AV439–AV441 (Supplementary Table 2). To induce the expression of the different ^*N*-pep^PBP1b constructs, o/n cultures were back diluted 1:500 and induced by growth in 0.2% L-arabinose and 250 µM IPTG in LB, at 30 °C. Cells were collected at OD_600_ = 0.3 and imaged on 2% agarose in LB pads using the same setup as described above. Cell length was determined in MicrobeJ^83^ and foci were detected using TrackMate^41^.

### Sequence and conservation analysis of PBP1b N-IDD

To assess amino acid sequence conservation of ^*N*-pep^PBP1b, we ran iterative HHM JackHMMER with default parameters on the EMBL-EBI web interface (https://www.ebi.ac.uk/Tools/hmmer/search/phmmer)^92^. The search was restricted to bacteria. Obtained hits (113) were imported and displayed using AnnoTree (v.1.2)^93^. Randomly picked sequences of ^*N*-pep^PBP1b were tested for interaction with FtsA using ColabFold (see above). In contrast, running the glycosyltransferase domain of PBP1b with the same parameters resulted in 22,750 hits distributed across the whole bacterial domain. Classical multiple sequence alignments (MSA) were carried out using Clustal Omega on the EMBL webserver (https://www.ebi.ac.uk/jdispatcher/msa/clustalo) using default parameters^94^. Predictions for intrinically disordered domains (IDDs) were calculated using the PrDOS server (https://prdos.hgc.jp/cgi-bin/top.cgi) with a 1% false positivity rate cut-off^95^. Protein structures were visualized using ChimeraX^77,96,97^. UniProt accession numbers for all tested proteins for in silico (ColabFold, MSA, IDDs prediction) analysis can be found in Supplementary Table 7.

### FtsZ treadmilling assay on supported lipid bilayers

#### Purification and labelling of proteins

Alexa Fluor 488-labelled FtsZ and FtsA were purified following previously established protocols^98,99^. ^*N*-pep^PBP1b (residues 1–64) peptide and an R6E derivative, modified with a C-terminal cysteine and an N-terminal 6× His tag, were obtained from Biomatik. Peptide labelling was performed using a modified version of a previously described method^99^. Briefly, lyophilised peptides were dissolved in ddH2O at a concentration of 1 mg ml^−1^ and reduced with a 20-fold molar excess of Tris(2-carboxyethyl)phosphine for 20 min at room temperature. Subsequently, a thiol-reactive sulfo-cyanine5-maleimide dye (Lumiprobe), dissolved in dimethyl sulfoxide, was added at a 5-fold molar excess and incubated for 3 h at room temperature. Labelled peptides were then dialysed overnight at 4 °C against reaction buffer (50 mM Tris-HCl pH 7.4, 150 mM KCl, 5 mM MgCl_2_). Excess unreacted dye was removed using a PD10 desalting column (Cytiva), after which peak fractions were collected, flash frozen in liquid nitrogen and stored at −80 °C.

#### Preparation of small unilamellar vesicles (SUVs)

Experiments were conducted using a lipid composition of 1,2-dioleoyl-*sn*-glycero-3-phospho-(1’-rac-glycerol) (DOPC) and 1,2-dioleoyl-*sn*-glycero-3-phospho-(1′-rac-glycerol) (DOPG) in a 2:1 molar ratio. Chloroform-dissolved lipids were combined in a glass vial, vortexed and dried under a stream of filtered N_2_ to form a thin, homogeneous film. Residual chloroform was removed by placing the vials under vacuum for 3 h. The lipid film was subsequently rehydrated in reaction buffer (50 mM Tris-HCl pH 7.4, 150 mM KCl, 5 mM MgCl_2_) for 30 min at 37 °C to achieve a final lipid concentration of 5 mM. The suspension was vigorously vortexed to form multilamellar vesicles, which were subjected to 8–10 freeze–thaw cycles using liquid N_2_. SUVs were generated by tip-sonicating the vesicle dispersion for 20 min, followed by centrifugation at 20,000 *g* for 5 min. The supernatant was collected, stored at 4 °C and used within 1 week.

#### Sample preparation

Glass coverslips (#1.5H) were cleaned using piranha solution (30% H_2_O_2_ mixed with concentrated H_2_SO_4_ at a 1:3 ratio) for 1 h, followed by extensive washing and sonication in ddH_2_O for 30 min. Cleaned coverslips were stored in ddH_2_O for up to 1 week. Before use, coverslips were dried with compressed air. Reaction chambers were assembled by affixing a 0.5-ml Eppendorf tube (with the conical end removed) to a coverslip using ultraviolet (UV) glue (Norland Optical Adhesive 63), followed by UV light exposure for 3 min.

Supported lipid bilayers (SLBs) were formed by diluting the SUV dispersion in reaction buffer to a final lipid concentration of 0.5 mM. SLB formation was induced by the addition of 5 mM CaCl_2_, followed by incubation at 37 °C for 15–20 min. Excess non-fused vesicles were removed by washing the surface 10 times with reaction buffer. SLBs were used immediately after preparation.

#### Total internal reflection microscopy

All in vitro experiments involving SLBs were performed on a Nikon Ti2E stand equipped with an iLas2 (GATACA) 360°/Ring/Azimuthal TIRF module and a ×100 NA 1.49 CFI Apochromat oil immersion objective. Fluorophores were excited using 488 nm and 640 nm laser lines from an Omicron LightHUB Ultra laser system. Emitted fluorescence was split using a TwinCam Cube 643 nm and further filtered with a 525/50 ET bandpass and a 635 nm longpass filter. Time series were recorded using Andor iXon Life 888 Back-Illuminated EMCCD cameras.

#### Peptide–filament interaction experiments

Self-organization of FtsA and FtsZ into treadmilling filament networks on SLBs was performed using FtsA (0.4 μM) and Alexa Fluor 488-labelled FtsZ (1.25 μM) in 100 μl of reaction buffer supplemented with 4 mM ATP and 4 mM GTP. To minimize photobleaching during imaging, the reaction buffer was supplemented with 30 mM D-glucose, 0.050 mg ml^−1^ glucose oxidase, 0.016 mg ml^−1^ catalase, 1 mM dithiothreitol and 1 mM Trolox.

To investigate the effect of ^*N*-pep^PBP1b peptide on the treadmilling filament network, FtsA and FtsZ were incubated until large-scale filamentous patterns emerged. The dynamic filament pattern was monitored for 15 min, with image acquisition at one frame per 2 s and an exposure time of 50 ms for both the 488-nm and 640-nm channels. During imaging, either ^*N*-pep^PBP1b WT or ^*N*-pep^PBP1b (R6E) variants (both labelled with cyanine 5, 0.4 μM) were introduced into the reaction chamber.

#### Image analysis

For data analysis, movies were imported into FIJI (ImageJ). All micrographs presented in this study were processed using the walking average plugin in ImageJ, averaging the signal of two consecutive frames to enhance visual clarity while preserving relative intensities over time. Look-up tables of PBP1b peptide micrographs were adjusted in Adobe Illustrator to ensure colour consistency. For intensity-over-time projections in Fig. 5d, time-lapse frames captured during sample addition were removed. The resulting data were then normalized to the maximum intensity value for individual replicates. For co-localization data analysis, Pearson’s correlation coefficient analysis was performed using numpy.corrcoef^100^ (Extended Data Fig. 7).

### Statistical analysis

All data measurements were plotted and analysed using GraphPad Prism 10 (v.10.2.2). In general, (log-) normal distribution was tested by using the Shapiro–Wilk test. For comparisons of two groups, significance was determined using two-tailed, unpaired Student’s *t*-test with Welch correction. One-way analysis of variance (ANOVA) was used for comparisons of more than two groups using the recommended post test for selected pairwise comparisons. *P* values less than 0.05 were considered statistically significant. Further details on statistical tests such as sample size and number of biological repeats are provided in figure legends. For co-localization data analysis, Pearson’s correlation coefficient analysis was performed using numpy.corrcoef^100^ (Extended Data Fig. 7).

### Reporting summary

Further information on research design is available in the Nature Portfolio Reporting Summary linked to this article.

## Supplementary information

Supplementary Information Supplementary Tables 1–7 and Videos 1–8 legends.

Reporting Summary

Supplementary Video 1 In situ architecture of sPG in wild-type *E. coli*. Cryo-electron tomogram of wild-type *E. coli*. Time-lapse series were acquired with a rate of 7 fps in the compressed format m4v for visualization purposes. Green, cyan and magenta layers indicate segmented IM, PG and OM, respectively. Ribosomes are shown in yellow. Scale bars, 100 nm.

Supplementary Video 2 In situ architecture of sPG in ∆ponB *E. coli*. Cryo-electron tomograms of ΔponB cells. Details as in Supplementary Video 1.

Supplementary Video 3 In situ architecture of sPG in ∆lpoB *E. coli*. Cryo-electron tomograms of ΔlpoB cells. Details as in Supplementary Video 1.

Supplementary Video 4 In situ architecture of sPG in ∆ponA *E. coli*. Cryo-electron tomograms of Δ*ponA* cells. Details as in Supplementary Video 1.

Supplementary Video 5 Time-lapse video of *E. coli* cells subjected to osmotic oscillations. Indicated strains were imaged in a microfluidic flow cell (CellAsic) in the presence of 1 µM SYTOX Green or 1 µM propidium iodide (αponB(R6E)). Osmotic oscillations were performed by switching media from LB to 0.5×LB0N ten times over a 42-min observation period. Images were acquired at 0.1 Hz. Individual phase (centre) and fluorescence (right) channels are shown in addition to a merged overlay (left). Representative examples for WT, ∆ponB, ∆ponB pNP146 (Para::sulA), ∆ponB pAV39 (Plac::ponB(R6E, M46L)), ∆lpoB and ∆ponA are sequentially shown. For further details, see Methods. Scale bar, 2 µm.

Supplementary Video 6 Side-by-side comparison of SPT experiments with *E. coli* cells expressing Halo fusions to the indicated PBP1b isoforms. STP trajectories of indicated Halo–PBP1b isoforms (labelled with 20 nM JF549) are coloured according to their mean track speed (blue = slow, red = fast). ZapA–sfGFP (false coloured in green) was used as a fiducial marker for the divisome and overlayed over a bright-field reference image. Images were recorded at 20 Hz and binned 2 × 2. For further details, see Methods. Scale bar, 0.5 µm.

Supplementary Video 7 FtsZ treadmilling assay in response to N-αPBP1b peptide addition. Alexa488 labelled FtsZ (1.25 µM) and unlabelled FtsA (0.4 μM) were reconstituted on supported lipid bilayers (SLBs) in the presence of 4 mM ATP/GTP and allowed to self-organize into treadmilling filaments and followed by TIRF microscopy at 0.5 Hz acquisition frame rate. At 2 min, 0.4 µM Cy5 labelled N-αPBP1b peptide was added to the reaction chamber. For further details, see Methods. Scale bar, 5 µm.

Supplementary Video 8 FtsZ treadmilling assay in response to N-αPBP1b(R6E) peptide addition. Alexa488 labelled FtsZ (1.25 µM) and unlabelled FtsA (0.4 μM) were reconstituted on SLBs in presence of 4 mM ATP/GTP and allowed to self-organize into treadmilling filaments and followed by TIRF microscopy at 0.5 Hz acquisition frame rate. At 2 min, 0.4 µM Cy5 labelled N-αPBP1b(R6E) peptide was added to the reaction chamber. For further details, see Methods. Scale bar, 5 µm.

Supplementary Data 1 Hits from AlphaFold multimer screen for PBP1b isoforms. For additional information, check https://private.predictomes.org/library/help.

## Source data

Source Data Fig. 1 Statistical source data.

Source Data Fig. 2 Statistical source data.

Source Data Fig. 3 Statistical source data.

Source Data Fig. 4 Statistical source data.

Source Data Fig. 6 Statistical source data.

Source Data Extended Data Fig. 1 Statistical source data.

Source Data Extended Data Fig.2 Statistical source data.

Source Data Extended Data Fig. 4 Statistical source data.

Source Data Extended Data Fig. 5 Statistical source data.

Source Data Extended Data Fig. 7 Statistical source data.

Source Data Extended Data Fig. 8 Unprocessed western blot.

Source Data Extended Data Fig. 8 Unprocessed western blot.

## Data Availability

The data, plasmids and strains that support the findings of this study are available from the corresponding authors by request. Representative tomograms are deposited in EMDB: EMD-27479 (wild-type), EMD-53351 (*∆ponB*), EMD-53357*(∆lpoB*) and EMD-53363 (*∆ponA*). Corresponding raw movie frames and stacks of tilt series are deposited as EMPIAR-11090 (wild type), EMPIAR-13502 (*∆ponB*), EMPIAR-13513 (*∆lpoB*) and EMPIAR-13512 (*∆ponA*), and will be released upon publication. Other data related to this manuscript (for example, AFM, light microscopy, growth curves and so on) can be found on Zenodo at 10.5281/zenodo.20841819 (ref. ^101^). Source data are provided with this paper.

## Code availability

Scripts used in this study were deposited on GitHub at https://github.com/NavarroVettiger/Navarro-et-al_2022 and https://github.com/virlyananda/EM-ImageProcessing.
