# Supplementary material for: The penicillin-binding protein PBP1b fortifies the Escherichia coli division site against osmotic rupture
Source: Nat Microbiol. 2026 Jul 3;11(8):2142–56. doi: 10.1038/s41564-026-02403-6 (PMC13423804; doi:10.1038/s41564-026-02403-6)
Supplement: Supplementary file 1 — Supplementary Tables 1–7 and Videos 1–8 legends. [file 41564_2026_2403_MOESM1_ESM.pdf]

nature microbiology

Supplementary information https://doi.org/10.1038/s41564-026-02403-6

The penicillin-binding protein PBP1b fortifies the *Escherichia coli* division site against osmotic rupture

In the format provided by the authors and unedited

Supplemental Material For:

The penicillin-binding protein PBP1b fortifies the *Escherichia coli* division site against osmotic rupture

Authors

Paula P. Navarro[1,2,3,4†*], Andrea Vettiger[1,2,†*], Roman Hajdu[5], Virly Y. Ananda[1,3], Alejandro de Tavares[1], Ernst W. Schmid[6], Johannes C. Walter[6,7], Martin Loose[5], Luke H. Chao[3,4] and Thomas G. Bernhardt[2,7*]

Affiliations

[1] Department of Fundamental Microbiology, Faculty of Biology and Medicine, University of Lausanne, Lausanne, Switzerland

[2] Department of Microbiology, Blavatnik Institute, Harvard Medical School, Boston, USA

[3] Department of Molecular Biology, Massachusetts General Hospital, Boston, USA

[4] Department of Genetics, Blavatnik Institute, Harvard Medical School, Boston, USA

[5] Institute of Science and Technology Austria (ISTA), Klosterneuburg, Austria

[6] Department of Biological Chemistry & Molecular Pharmacology, Blavatnik Institute, Harvard Medical School, Boston, USA

[7] Howard Hughes Medical Institute, Harvard Medical School, Boston

[†] These authors contributed equally, and sequence was determined alphabetically.

* To whom correspondence should be addressed

Thomas G. Bernhardt

e-mail: thomas_bernhardt@hms.harvard.edu

Paula P. Navarro

e-mail: paula.navarro@unil.ch

Andrea Vettiger

e-mail: andrea.vettiger@unil.ch

Keywords

Bacterial cell division, Cell wall remodeling, Class A penicillin binding proteins, Cryo-electron tomography, Cryo-focused ion beam milling, Fluorescence live-cell microscopy, Atomic force microscopy, AlphaFold screening

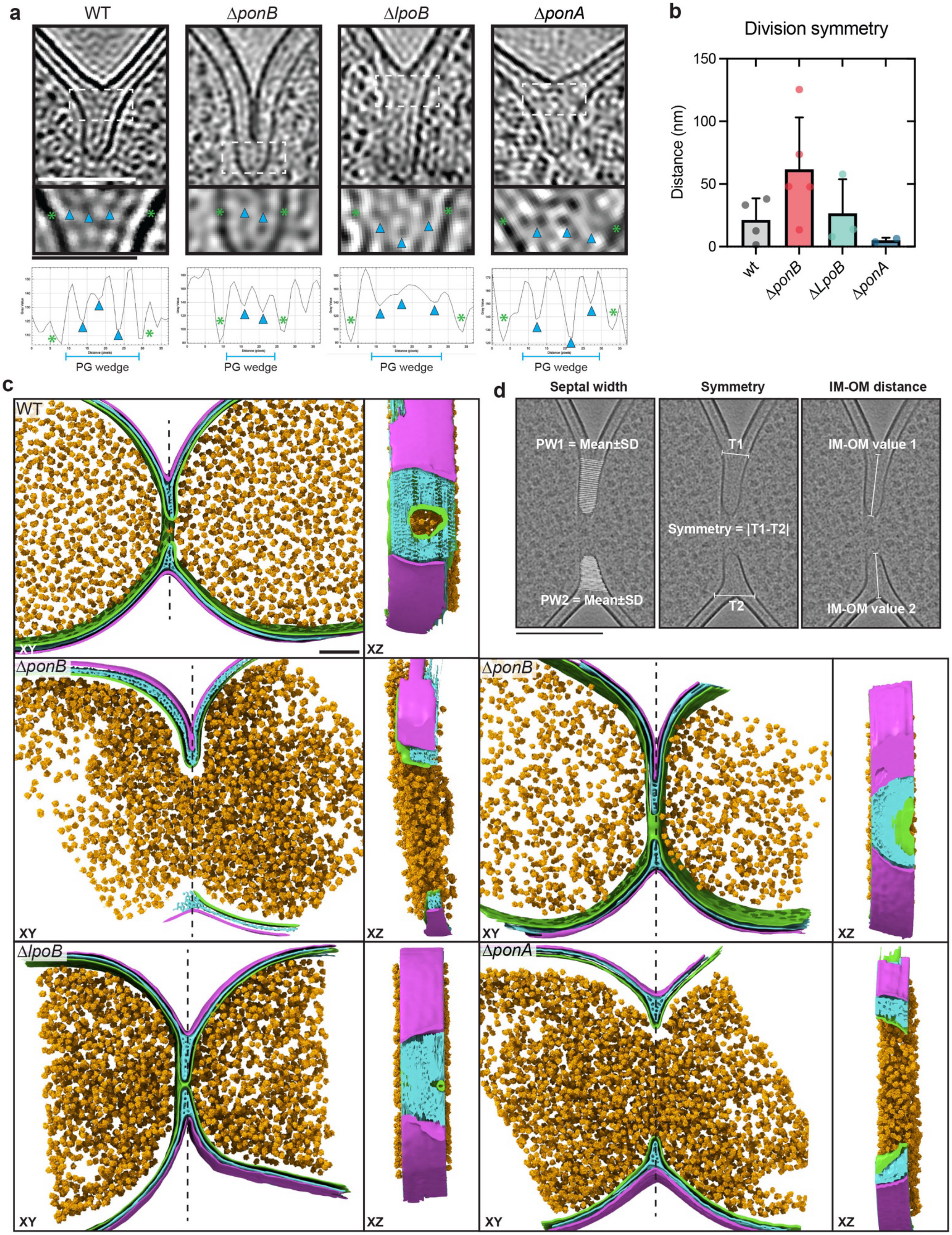

Extended Data 1: Detailed architecture of the division site by *in situ* cryo-ET. **(a)** Summed, projected central slices of low-passed filtered cryo-electron tomograms shown in Figure 1. White dashed box

indicates the region of corresponding zoom-in images of the typical sPG wedge-containing region below. Bottom row shows corresponding normalized grey-scale profiles of the sPG wedge region. Green asterisks indicate IM regions and blue arrows indicate PG. Note the reduced number of detectable PG densities within the septum of the Δ*ponB* cell. **(b)** Bar graph showing the difference in nm between the length of the tangent line to the OM tip from IM to IM of both sides of the division site. This denotes division site symmetry. **(c)** 3D surface segmentation renderings of IM (green), PG (cyan), OM (magenta) and ribosomes (yellow) are shown as top view (XY plane) and side view (XZ plane). Dashed line indicates the surface cut made to show a cross view of the PG architecture at the division site in the corresponding side views (XZ). **(d)** Example of measurements performed in Fig. 1d-e and Fig. S1b in a summed projected central slice of dividing wild-type *E.coli*. N values for each strain are: N = 5 (WT); 5 (Δ*ponB*); 3 (Δ*lpoB*) and 2 (Δ*ponA*). Scale bars = 100 nm.

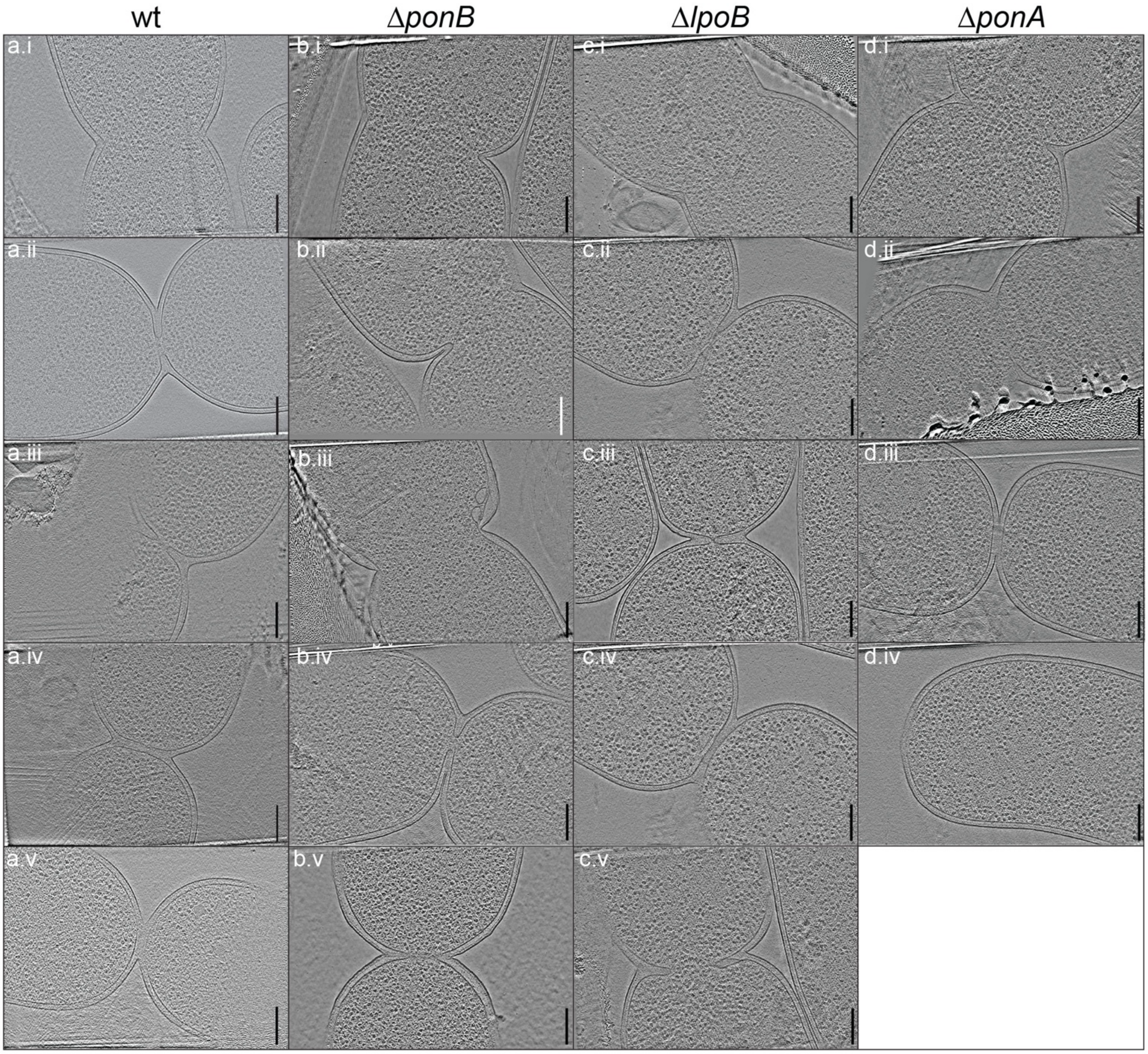

Extended Data 2: Gallery of cryo-ET data of dividing *E. coli* cells. Three dimensional slices visualizing the division site and pole of the indicated strains. Scale bar = 200 nm. A complete overview of number of tomograms (number of cells imaged) and data acquisition is reported in **Table S1**. WT data from[1]. Scale bars = 200 nm.

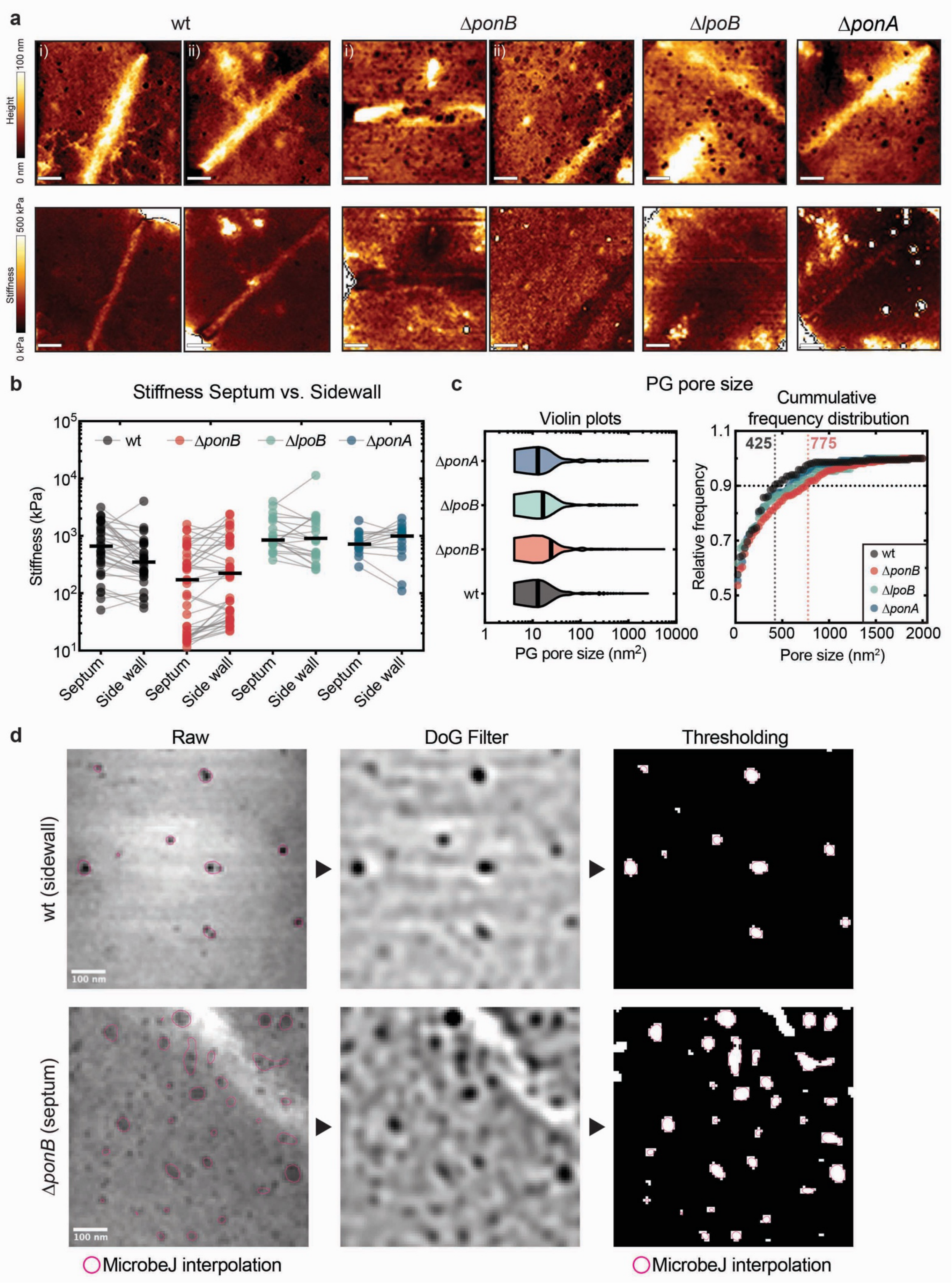
a
wt
ΔponB
ΔlpoB
ΔponA
i)
ii)
Height
0 nm
100 nm
Stiffness
0 kPa
500 kPa
b
Stiffness Septum vs. Sidewall
Stiffness (kPa)
Septum
Side wall
c
PG pore size
Violin plots
PG pore size (nm2)
Cummulative frequency distribution
Relative frequency
Pore size (nm2)
425
775
d
Raw
DoG Filter
Thresholding
wt (sidewall)
ΔponB (septum)
100 nm
MicrobeJ interpolation

52

Extended Data 3: Septal ultrastructure and stiffness characterization of isolated PG sacculi by AFM. (a) Additional examples of high-resolution (1 x 1 µm, 7.81nm pixel size) height (top) and Young's modulus (stiffness, bottom) maps of indicated strains. Scale bar = 200 nm. **(b)** Absolute quantification of septal and sidewall stiffness of indicated strains. Lines connect measurements from the same sacculi. N sacculi for each strain from 3 biological replicates were measured: N = 37 (WT); 40 *(ΔponB*); 21 (Δ*lpoB*); 15 (Δ*ponA*). **(c)** Violin plots (left) and cumulative frequency distribution (right) of PG pore size as determined from high-resolution height images (see Methods). Solid line represents median pore size (violin plots), dotted lines and numbers indicates the 90th percentile of the cumulative distribution corresponding to 425 nm (WT) and 775 nm (Δ*ponB*). In other words, 90% of the peptidoglycan pores are ≤ 425 nm^2 or ≤ 775 nm^2 in surface area, respectively. Numbers of pores measured for each strain were: 254 (WT); 508 (Δ*ponB*); 144 (Δ*lpoB*); 147 (Δ*ponA*). **(d)** Example of image analysis procedure for quantification of PG pores. A difference of gaussian (DoG) filter was applied prior to using thresholding (default, FIJI) and interpolation in MicrobeJ (outlined in magenta).

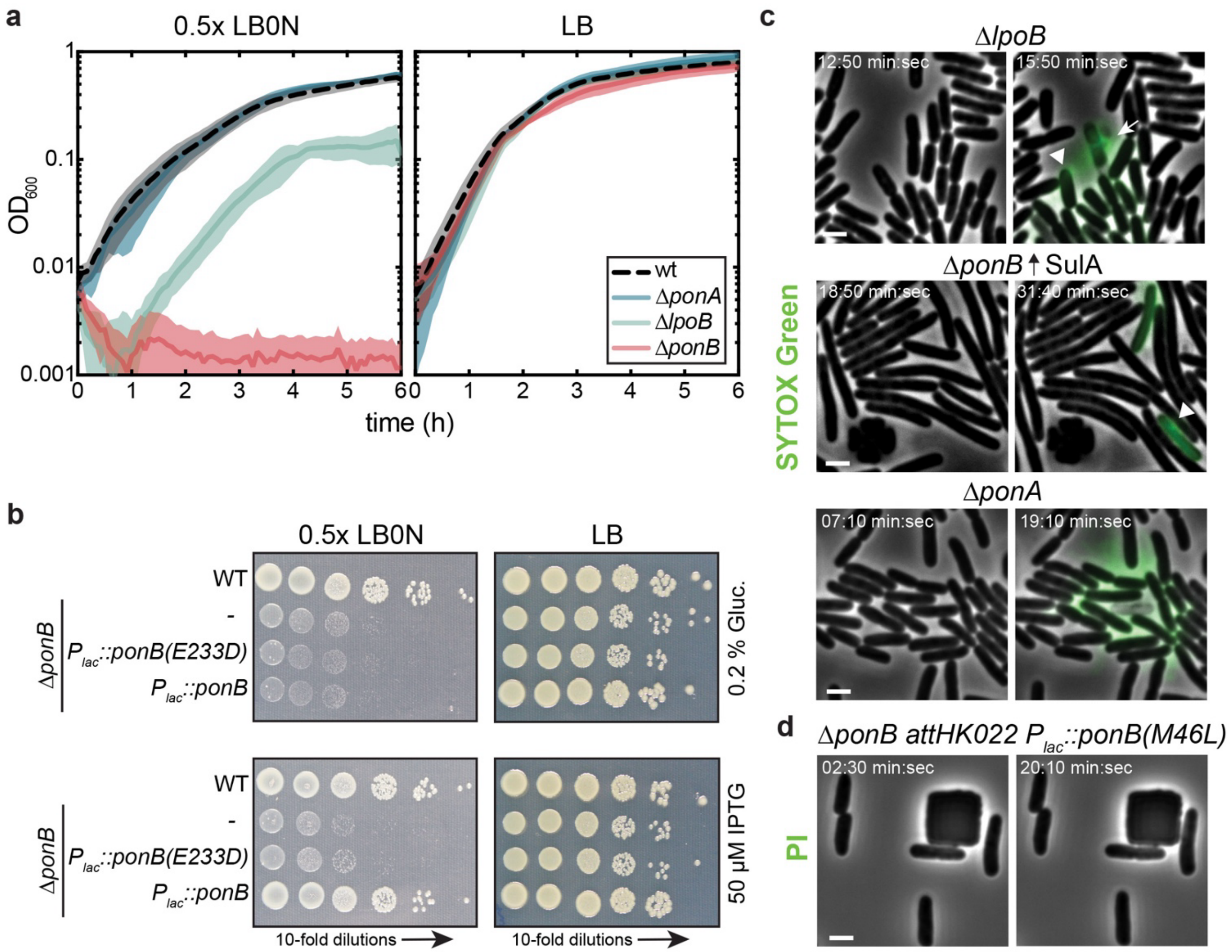

Extended Data 4: Cells lacking PBP1b are sensitive to osmotic shifts. **(a)** Growth of the indicated strains was assessed on a plate reader at 42°C in LB or 0.5xLB0N, respectively. Day cultures of the indicated strains were back diluted to OD_{600} = 0.01 and growth was followed for 6h. The line represents mean ± one SD of the absorbance value obtained from three biological replicates. **(b)** Representative image of a bacterial viability assay. Cells were grown on LB or 0.5xLB0N agar supplemented with either 0.2% glucose or 50 µM IPTG at 42°C as indicated. Strains deleted for *ponB* carry indicated single-copy alleles at the attHK022 phage integration site. Data is representative for 3 biological replicates. **(c)** Representative images of additional mutants imaged in response to osmotic oscillations (see Fig. 3b). In the second set of panels filamentation was induced by expression of *sulA* from pNP146 for 20 min prior exposure to the first osmotic shock. White arrows point to sites of septal lysis events, and arrowheads highlight polar lysis events. **(d)** Representative image of the indicated strain quantified in Figure 6b.

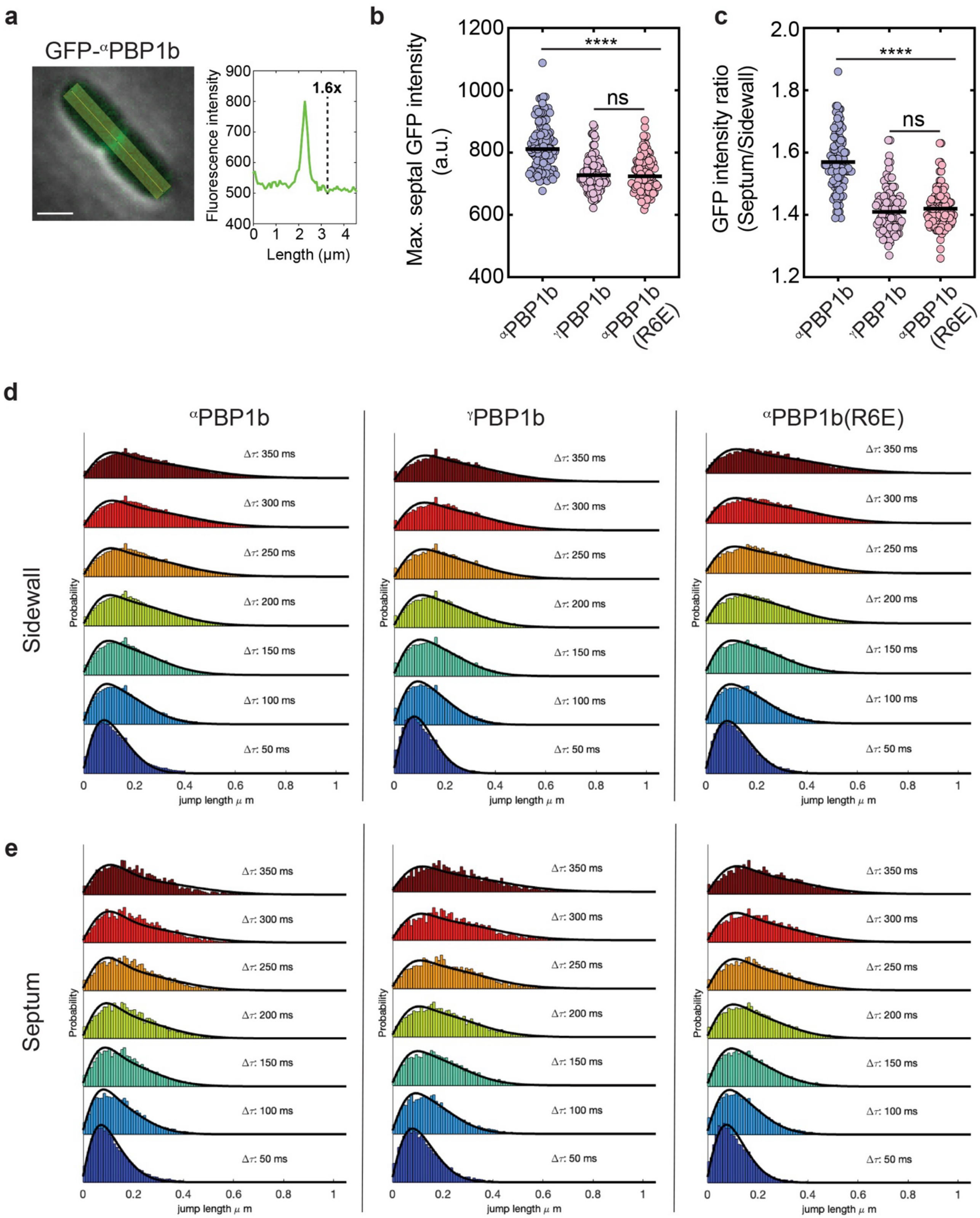

Extended Data 5: Preferential localization and activity of different PBP1b fusions. (a) Representative image of a cell expressing GFP-αPBP1b (AV266) induced with 50µM IPTG. In constricting cells, GFP intensity was measured as a function of cell length and averaged over 10 pixels (0.65 µm). Scale bar = 1 µm. **(b)** Maximal septal GFP intensity, as well as the **(c)** ratio between the maximal (at the septum) and

minimal (along the sidewall) intensity were plotted. Line represents median and significance was tested using one-way ANOVA with Tuckey's posttest. Stars indicate significant differences, ns = non-significant. $^{\alpha}$PBP1b vs $^{\gamma}$PBP1b: $p = 1.044 \times 10^{-12}$, $^{\alpha}$PBP1b vs $^{\alpha}$PBP1b(R6E): $p = 1.041 \times 10^{-12}$, $^{\gamma}$PBP1b vs $^{\alpha}$PBP1b(R6E): $p = 0.9533$; $^{\alpha}$PBP1b (N = 109), $^{\gamma}$PBP1b (N = 100), $^{\alpha}$PBP1b(R6E) (N = 105). For the comparison of septal to side wall GFP intensities Kurskal-Wallis with Dunn's posttest was applied. **** $p < 1 \times 10^{-15}$, ns = $p > 0.9999$. **(d-e)** Quantification of the stationary and diffusive fraction of single particle trajectories. Observed and fit distributions of particle jump lengths at the (d) side wall or (e) septum over eight steps with a 20 Hz acquisition frame rate (Δt = 50ms) each, obtained using the Spot-On tool[42].

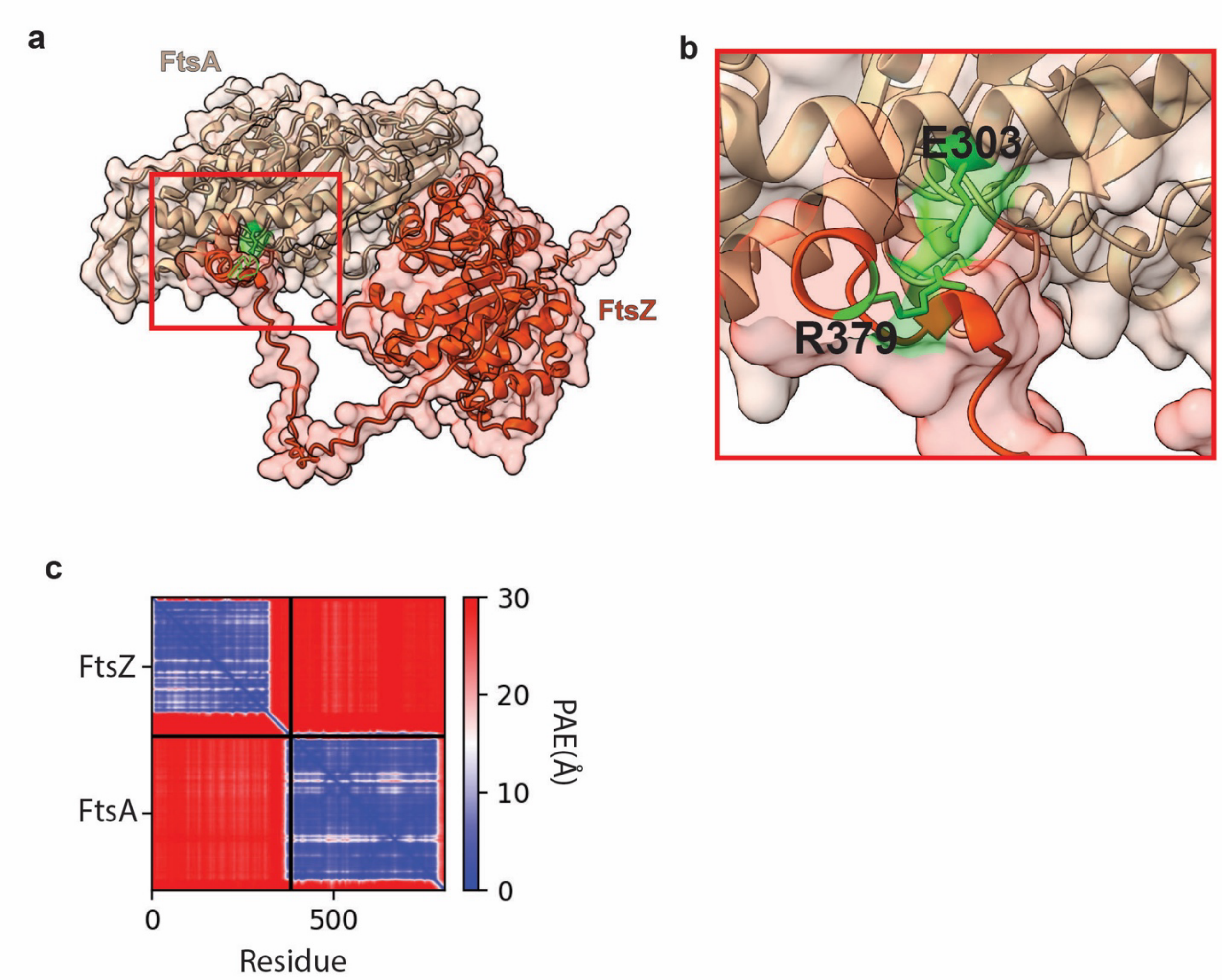

Extended Data 6: The FtsZ-FtsA interaction interface overlaps with the predicted $^{\text{N-pep}}$PBP1b-FtsA interface. (a) Predicted structure of an *E. coli* FtsA (salmon)-FtsZ (red) complex. Red box highlights the magnified region shown in (b). **(b)** Magnified image of (a) showing the interaction between FtsA E303 residue and FtsZ R379 residue (green). **(c)** Predicted alignment error in Å of all residues against all residues. Low error (blue) corresponds to well-defined relative domain positions. The binding position identified in the model mirrors that identified in the *Thermotoga* structure[2].

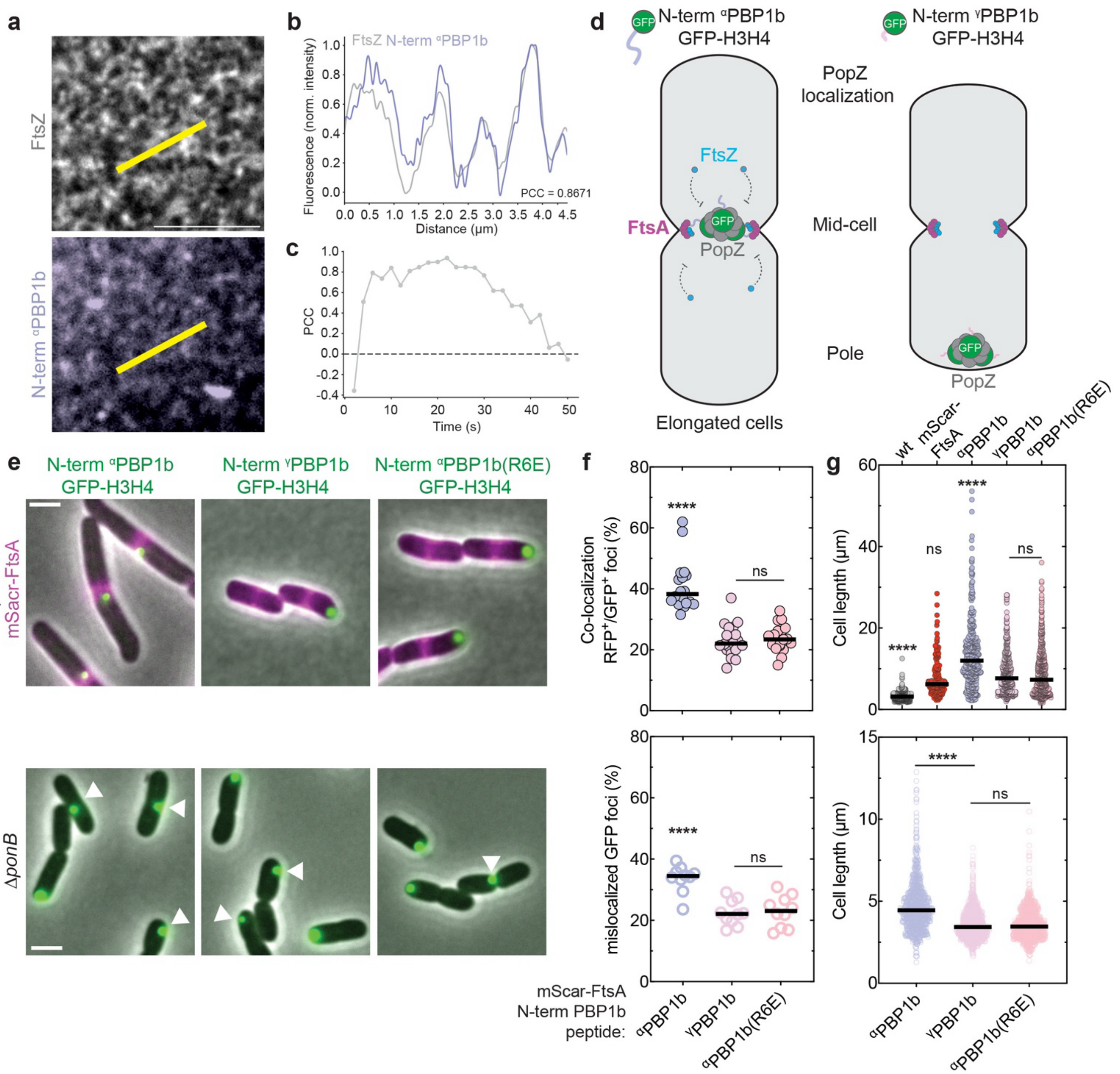

Extended Data 7. Transient colocalization of $^{N\text{-}pep}$PBP1b and FtsZ filaments *in vitro* and polar relocalization of N-term PBP1b constructs. (a) Micrograph of a single frame shortly after addition of $^{N\text{-}pep}$PBP1b. **(b)** Fluorescent intensity profiles from AF488-FtsZ and Cy5-$^{N\text{-}pep}$PBP1b corresponding to line ROI indicated in panel a. Pearson's correlation coefficient (PCC) analysis was performed using numpy.corrcoef[100] using the standard 2x2 correlation matrix of interpolated intensity profiles. **(c)** PCC values over time between normalized intensities (maximum intensity normalization) of AF488-FtsZ and Cy5- $^{N\text{-}pep}$PBP1b in ROI from panel a, t = 0 s marks addition of Cy5- $^{N\text{-}pep}$PBP1b. **(d)** Schematic overview of localization of N-terminal PBP1b peptide fusions to GFP and the PopZ-interacting peptide H3H4 in cells producing an mScarlet-FtsA fusion and heterologously producing *Caulobacter crescentus* PopZ. This assay is based on the same principle as the POLAR two-hybrid assay[45]. **(e)** Top: Representative image of cells co-expressing PopZ with either $^{N\text{-}pep}$PBP1b (AV439) fused to GFP and H3H4 or similar fusions with the N-terminal peptide of $^{\gamma}$PBP1b (AV440) or $^{N\text{-}pep}$PBP1b(R6E) (AV441). The GFP fusions were produced from the P_{ara} promoter induced with 0.2 % arabinose. These cells also express mScar-FtsA under P_{lac} promoter control expressed from the *att*HK022 site induced with 250 µM IPTG. Bottom: cells expressing $^{N\text{-}pep}$PBP1b (AV442), $^{\gamma}$PBP1b (AV443) or $^{N\text{-}pep}$PBP1b(R6E) (AV444) in absence of mScar-FtsA. Arrowhead indicates mislocalized GFP foci. Scale bars = 2 µm. **(f)** Top: quantification of GFP signals overlapping with

mScar-FtsA (RFP) at division sites. Significant differences relative to $^{\alpha}$PBP1b were determined using one-way ANOVA with Dunnett's posttest. Stars indicate significance, ns = non-significant. ($^{\alpha}$PBP1b vs $^{\gamma}$PBP1b: $p = 6.238 \times 10^{-12}$, $^{\alpha}$PBP1b vs $^{\alpha}$PBP1b(R6E): $p = 3.0736 \times 10^{-11}$, $^{\gamma}$PBP1b vs $^{\alpha}$PBP1b(R6E): $p = 0.9342$). N = total number of images analyzed: $^{\alpha}$PBP1b (N = 18), $^{\gamma}$PBP1b (N = 20), $^{\alpha}$PBP1b(R6E) (N = 19). Bottom: quantification of mislocalized (non-polar) GFP foci; ($^{\alpha}$PBP1b vs $^{\gamma}$PBP1b: $p = 2.0364 \times 10^{-5}$, $^{\alpha}$PBP1b vs $^{\alpha}$PBP1b(R6E): $p = 3.9258 \times 10^{-5}$, $^{\gamma}$PBP1b vs $^{\alpha}$PBP1b(R6E): $p = 0.9671$).$^{\alpha}$PBP1b (N = 10), $^{\gamma}$PBP1b (N = 10), $^{\alpha}$PBP1b(R6E) (N = 10). **(g)** Top: quantification of cell length in indicated strains, wt and parental mScar-FtsA. Kurskal-Wallis with Dunn's posttest was applied. Stars indicate significance, ns = non-significant. (WT vs mScar-FtsA: $p < 1 \times 10^{-15}$, WT vs $^{\alpha}$PBP1b: $p < 1 \times 10^{-15}$, WT vs $^{\gamma}$PBP1b: $p < 1 \times 10^{-15}$, WT vs $^{\alpha}$PBP1b(R6E): $p < 1 \times 10^{-15}$, mScar-FtsA vs $^{\alpha}$PBP1b: $p = 1.4491 \times 10^{-9}$, mScar-FtsA vs $^{\gamma}$PBP1b: $p > 0.9999$, mScar-FtsA vs $^{\alpha}$PBP1b(R6E): $p > 0.9999$, $^{\alpha}$PBP1b vs. $^{\gamma}$PBP1b: $p = 3.111 \times 10^{-11}$, $^{\alpha}$PBP1b vs. $^{\alpha}$PBP1b(R6E): $p = 5 \times 10^{-15}$, $^{\gamma}$PBP1b vs. $^{\alpha}$PBP1b(R6E): $p > 0.9999$). N = total number of cells analyzed: wt (N=540), mScar-FtsA (N = 155), $^{\alpha}$PBP1b (N = 326), $^{\gamma}$PBP1b (N = 442), $^{\alpha}$PBP1b(R6E) (N = 555). Bottom: quantification of cell length in indicates strains, ($^{\alpha}$PBP1b vs. $^{\gamma}$PBP1b: $p\ 1 \times 10^{-15}$, $^{\alpha}$PBP1b vs. $^{\alpha}$PBP1b(R6E): $p = 5 \times 10^{-15}$, $^{\gamma}$PBP1b vs. $^{\alpha}$PBP1b(R6E): $p > 0.9999$). $^{\alpha}$PBP1b (N = 936), $^{\gamma}$PBP1b (N = 1126), $^{\alpha}$PBP1b(R6E) (N = 976).

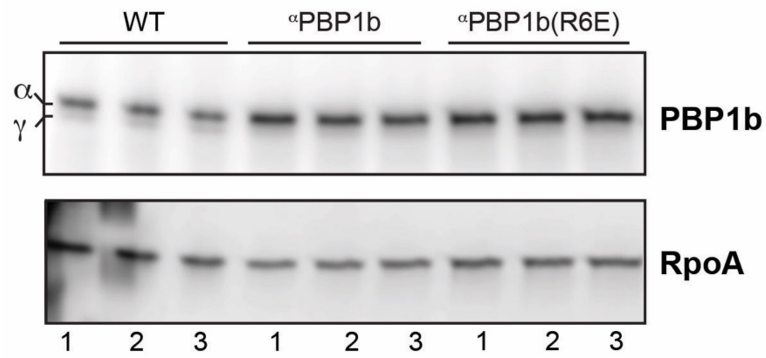

Extended Data 8: PBP1b isoforms are expressed at similar levels to WT. Protein levels were assessed by Western blot using polyclonal rabbit anti-PBP1b serum[3] and anti-RpoA mouse monoclonal antibody Samples were from WT cells expressing native PBP1b and Δ*ponB* cells expressing the indicated $^{\alpha}$PBP1b variant at the same induction levels used for complementation experiments. RpoA served as a loading control. Molecular weight for $^{\alpha}$PBP1b is 94.2 kDa (upper band) and for $^{\gamma}$PBP1b 88.9 kDa (lower band), respectively. The molecular weight of RpoA is 36.5 kDa. Protein sample from three biological replicated are displayed.

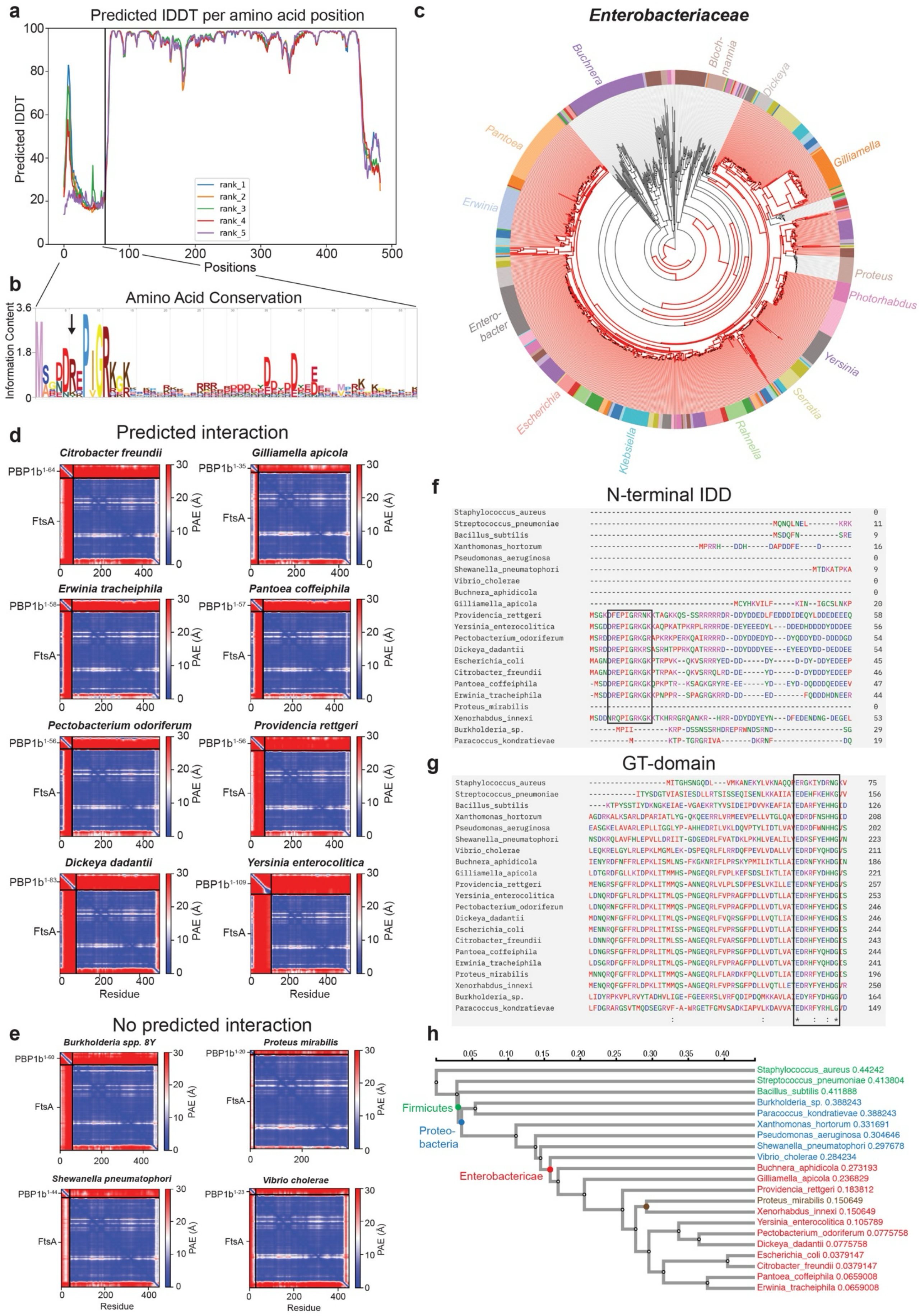
a
Predicted lDDT per amino acid position
Predicted lDDT
Positions
rank_1
rank_2
rank_3
rank_4
rank_5
b
Amino Acid Conservation
Information Content
c
Enterobacteriaceae
Buchnera
Bloch-mannia
Dickeya
Gilliamella
Pantoea
Erwinia
Proteus
Photorhabdus
Entero-bacter
Yersinia
Serratia
Escherichia
Klebsiella
Rahnella
d
Predicted interaction
Citrobacter freundii
Gilliamella apicola
Erwinia tracheiphila
Pantoea coffeiphila
Pectobacterium odoriferum
Providencia rettgeri
Dickeya dadantii
Yersinia enterocolitica
FtsA
PAE (Å)
Residue
e
No predicted interaction
Burkholderia spp. 8Y
Proteus mirabilis
Shewanella pneumatophori
Vibrio cholerae
f
N-terminal IDD
g
GT-domain
h
Firmicutes
Proteo-bacteria
Enterobactericae
Staphylococcus_aureus 0.44242
Streptococcus_pneumoniae 0.413804
Bacillus_subtilis 0.411888
Burkholderia_sp. 0.388243
Paracoccus_kondratievae 0.388243
Xanthomonas_hortorum 0.331691
Pseudomonas_aeruginosa 0.304646
Shewanella_pneumatophori 0.297678
Vibrio_cholerae 0.284234
Buchnera_aphidicola 0.273193
Gilliamella_apicola 0.236829
Providencia_rettgeri 0.183812
Proteus_mirabilis 0.150649
Xenorhabdus_innexi 0.150649
Yersinia_enterocolitica 0.105789
Pectobacterium_odoriferum 0.0775758
Dickeya_dadantii 0.0775758
Escherichia_coli 0.0379147
Citrobacter_freundii 0.0379147
Pantoea_coffeiphila 0.0659008
Erwinia_tracheiphila 0.0659008

Extended Data 9: Conservation analysis of $^{N\text{-}pep}$PBP1b and its potential interaction with FtsA. (a) Predicted local distance difference test of $^{N\text{-}pep}$PBP1b and FtsA. **(b)** Amino acid conservation of $^{N\text{-}pep}$PBP1b sequence as identified by JackHMMER. Black arrow indicates R6. **(c)** Hits (in red) from JackHMMER[4] search were visualized on a tree displaying the Enterobacteriaceae family using AnnoTree v1.2[5]. Closely related species that were not identified were added subsequently manually. **(d)** Additional examples of high-confidence predicted $^{N\text{-}pep}$PBP1b-FtsA complexes in distantly related Enterobacteriaceae. **(e)** AlphaFold predictions of $^{N\text{-}pep}$PBP1b-FtsA complexes using $^{N\text{-}pep}$PBP1b from Enterobacteriaceae family members not identified in the JackHMMER search as homologues (e.g. *Proteus mirabilis*) or PBP1b proteins found in other proteobacteria outside the Enterobacteriaceae family. Multiple sequence alignment (MSA) of full-length PBP1b proteins show amino acid conservation among distantly related bacteria for the **(f)** $^{N\text{-}pep}$PBP1b and the **(g)** catalytic GT domain. Black box highlights conserved amino acid sequence in a (sub)set of samples. **(h)** Phylogenetic tree reconstructed for MSA. Note the absence of $^{N\text{-}pep}$PBP1b sequence conservation in *P. mirabilis* (brown) (see also MSA in f).

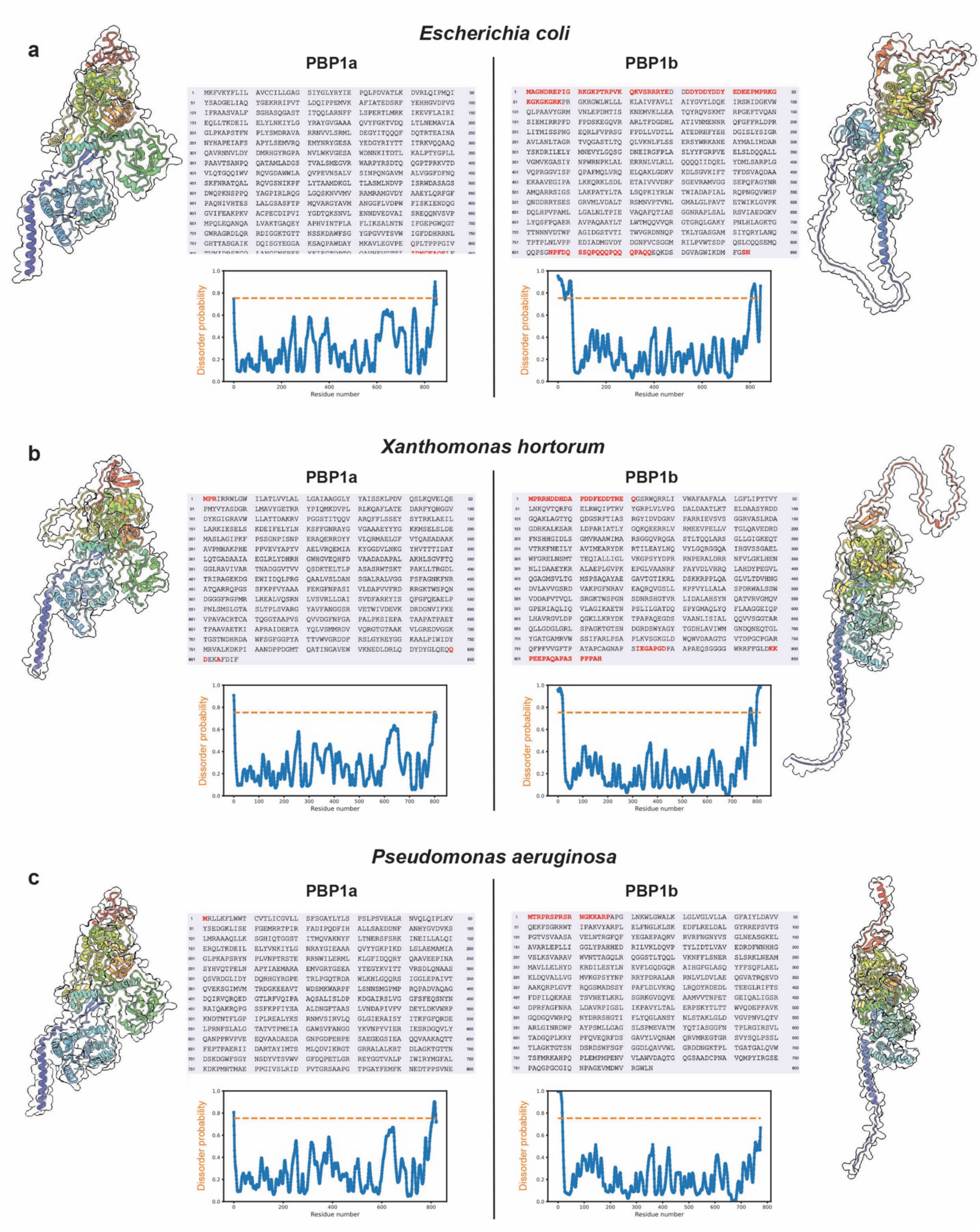

Extended Data 10: IDD domain prediction on aPBPs. AlphaFold and PrDOS highlights the presence of an on average 20-60 amino acid long N-terminal intrinsically disordered domain on PBP1b homologues which is absent on PBP1a homologues. PrDOS output displays predicted (< 1% false positivity cutoff) disordered domains in red on the amino acid sequence. Protein structures were represented in ChimeraX and are rainbow colored depending on amino acid position from their N-terminus (blue) to C-terminus (blue). Representative images for **(a)** *E. coli* (Enterobacteriaceae), **(b)** *Xanthomonas hortorum* (Enterobacterales), and **(c)** *Pseudomonas aeruginosa* (Pseudomonata) are displayed.

Table S1. Summary of data acquisition and image processing for cryo-ET data in this study.

*Data from *Navarro, Vettiger et al., 2022*[1].
Differences on pixel size reflect acquisition of data using different microscopes.

Sample		WT*	Δ*ponB*	Δ*lpoB*	Δ*ponA*
**Cryo-FIB milling**	**Microscope**	Aquilos Cryo-FIB, FEI – Thermo Fisher Scientific	Aquilos 1/2 Cryo-FIB, FEI – Thermo Fisher Scientific	Aquilos 2 Cryo-FIB, FEI – Thermo Fisher Scientific	Aquilos 2 Cryo-FIB, FEI – Thermo Fisher Scientific
**Acquisition settings**	**Microscope**	Titan Krios Gi3 FEI, Thermo Fisher Scientific	Titan Krios Gi3 FEI, Thermo Fisher Scientific	Titan Krios Gi3 FEI, Thermo Fisher Scientific	Titan Krios Gi3 FEI, Thermo Fisher Scientific
	**Voltage (KeV)**	300	300	300	300
	**Detector**	Gatan K3 IS	Gatan K3 IS	Gatan K3 IS	Gatan K3 IS
	**Energy filter**	Gatan BioQuantum K3	Gatan BioQuantum K3	Gatan BioQuantum K3	Gatan BioQuantum K3
	**Slit width (eV)**	20	20	20	20
	**Super-resolution mode**	Yes	Yes	Yes	Yes
	**Å/pixel**	2.565/2.758	2.758/2.704	2.704	2.704
	**Defocus (µm)**	-3.5 to -5.0	-3.5 to -5.0	-3.5 to -5.0	-3.5 to -5.0
	**Acquisition scheme**	-70/70, 2°, Dose-symmetric	-70/70, 2°, Dose-symmetric	-70/70, 2°, Dose-symmetric	-70/70, 2°, Dose-symmetric
	**Total dose**	~90 - 120	~90 - 180	~90 - 180	~90 - 120
	**Dose rate (e-/Å/sec)**	~ 1.5 - 3	~ 1.5 - 3	~ 1.5 - 3	~ 1.5 - 3
	**Frame number**	4 - 6	4 - 6	4 - 6	4 - 6
	**Number of tomograms**	22	27	11	4
**Image processing**	**Frame alignment and DW**	*framealign*, IMOD[6]	*framealign*, IMOD[6]	*framealign*, IMOD[6]	*framealign,* IMOD[6]
	**Tilt series alignment**	IMOD/*Dynamo*[7,8]	IMOD[7]	IMOD[7]	IMOD[7]
	**WBP**	IMOD[6]	IMOD[6]	IMOD[6]	IMOD[6]
	**Filtering**	Cryo-CARE[9]/Amira	Cryo-CARE[9]/Amira	Cryo-CARE[9]/Amira	Cryo-CARE[9]/Amira
	**3D-segmentation**	Amira/MemBrain[10,11]	Amira/MemBrain[10,11]	Amira/MemBrain[10,11]	Amira/MemBrain[10,11]
	**3D-rendering**	ChimeraX/ArtiaX[12,13]	ChimeraX/ArtiaX[12,13]	ChimeraX/ArtiaX[12,13]	ChimeraX/ArtiaX[10,11]

Table S2. Strains used in this study.

Strain	Genotype	Source/Reference
TB28	*rph1 ilvG rfb-50 ΔlacIZYA<>frt*	[14]
TU121	*TB28 ΔponA<>frt*	[3]
TU122	*TB28 ΔponB<>frt*	[3]
CB26	*TB28 ΔlpoB<>frt*	[3]
AV255	*TB28 (pAV13 P_{lac}::halo-$^{\alpha}$ponB(M46L))*	
AV258(attHKAV13)	*TU122 (P_{lac}::halo-$^{\alpha}$ponB(M46L))*	P1 AV255 (attHKAV13) x TU122
AV262	*TB28 (pAV15 P_{lac}::msfgfp-$^{\alpha}$ponB(M46L))*	
AV263	*TB28 pAV16 P_{lac}::ponB(E233D)*	
AV264(attHKHC942)	*TU122 (P_{lac}::msfgfp-$^{\gamma}$ponB(46-844))*	P1 HC576 (attHKHC942)[15]x TU122
AV265(attHKHC949)	*TU122 (P_{lac}::halo-$^{\gamma}$ponB(46-844))*	P1 HC576 (attHKHC949)[15] x TU122
AV266(attHKAV15)	*TU122 (pAV15 P_{lac}::msfgfp-$^{\alpha}$ponB(M46L))*	P1 AV262 (attHKAV15) x TU122
AV267(attHKAV16)	*TU122 (pAV16 P_{lac}::ponB(E233D))*	P1 AV263 (attHKAV16) x TU122
AV269	*TB28 ΔponB<>frt, pNP146*	
AV276(attHKAV13)	*AV258 zapA-sfgfp cat*	P1 (HC261)[16] x AV258
AV277(attHKHC949)	*AV265 zapA-sfgfp cat*	P1 (HC261)[16] x AV265
AV280	*TU122, pTB102*	
AV308(attHKAV12)	*AV280 (P_{lac}::ponB)*	CRIM vector was integrated using pTB102
AV347(attHKAV35)	*AV280, (P_{lac}::msfgfp-$^{\alpha}$ponB(R6E, M46L))*	CRIM vector was integrated using pTB102
AV352(attHKAV39)	*AV280, (P_{lac}::$^{\alpha}$ponB(R6E, M46L))*	CRIM vector was integrated using pTB102
AV404(attHKLM01)	*AV280, (P_{lac}::halo-$^{\alpha}$ponB(R6E, M46L))*	CRIM vector was integrated using pTB102
AV406(attHKLM01)	*AV404, zapA-sfgfp cat*	P1 (HC261)[16] x AV404
RA006	*TU122, (P_{lac}::mScar-ftsA)*	CRIM vector was integrated using pTB102
AV439	*RA006, pAV48*	
AV440	*RA006, pAV49*	
AV441	*RA006, pAV50*	
AV442	*TU122, pAV48*	
AV443	*TU122, pAV49*	
AV444	*TU122, pAV50*	
DH5αλpir	*F^- endA1 glnV44 thi-1 recA1 relA1 gyrA96 deoR nupG Φ80dlacZΔM15 Δ(lacZYA-argF)U169, hsdR17(r_K^- m_K^+), λpir*	[17]

Table S3. Plasmids used in this study.

Plasmid	Genotype[a]	ori	Source/Reference[b]
pTB102	*cat, intHK022*	pSC101ts	[14]
pNP146	*tetAR* P_{ara}*::sulA*	colE1	[18]
pHC942	*attHK022 tetAR* $lacI^q$ P_{lac}::*msfgfp-*$^{\gamma}$*ponB(46-844)*	R6K	[15]
pHC949	*attHK022 tetAR* $lacI^q$ P_{lac}::*halo-*$^{\gamma}$*ponB(46-844)*	R6K	[15]
pAV12	*attHK022 tetAR* $lacI^q$ P_{lac}*::ponB*	R6K	This study
pAV13	*attHK022 tetAR* $lacI^q$ P_{lac}*:: halo-*$^{\alpha}$*ponB(M46L)*	R6K	This study
pAV15	*attHK022 tetAR* $lacI^q$ P_{lac}*:: msfgfp-*$^{\alpha}$*ponB(M46L)*	R6K	This study
pAV16	*attHK022 tetAR* $lacI^q$ P_{lac}*::ponB(E233D)*	R6K	This study
pAV27	*attHK022 tetAR* $lacI^q$ P_{lac}*::* $^{\alpha}$*ponB(M46L)*	R6K	This study
pAV35	*attHK022 tetAR* $lacI^q$ P_{lac}*:: msfgfp-*$^{\alpha}$*ponB(R6E, M46L)*	R6K	This study
pAV39	*attHK022 tetAR* $lacI^q$ P_{lac}*::* $^{\alpha}$*ponB(R6E, M46L)*	R6K	This study
pAV41	*attHK022 tetAR* $lacI^q$ P_{lac}*::mScar-ftsA*	R6K	This study
pHCL149	P_{ara}*:: popZ H3H4-msfGFP camR*	colE1	[19]
pAV48	P_{ara}*:: popZ* $^{\alpha}$*ponB(1-63) -msfGFP-H3H4 camR*	colE1	This study
pAV49	P_{ara}*:: popZ* $^{\gamma}$*ponB(46-63)-msfGFP-H3H4 camR*	colE1	This study
pAV50	P_{ara}*:: popZ* $^{\alpha}$*ponB(R6E 1-63)-msfGFP-H3H4 camR*	colE1	This study
pLM001	*attHK022 tetAR* $lacI^q$ P_{lac}*:: halo-*$^{\alpha}$*ponB(R6E, M46L)*	R6K	This study
pCP20	*FLP, cat, bla*	repA101ts	[20]

Table S4. Primers used in this study.

Primer Name	Sequence (5'-3')[a]	Use
ponB_XbaI-Fw<br>ponB_BmtI-rev	tccccTCTAGAtttaagaaggagatatacatatgg ccgggaatgaccgcgagc<br>atgcaGCTAGCtcgaaacgctggcgggttcgca agc	Amplification of ponB from chromosome and cloning into pHC949 using XbaI/BmtI to generate pAV12
KLD_ponBE233D_Fw<br>KDL_ponBE233D_rev	tggcgacaCaagaccgtcatttttac<br>gcaaagtatccaccagcaaatccgg	Generation of E233D mutation using KLD kit using pAV12 for the generation of pAV16
ponB-alpha-BamHI_FW<br>ponB-NheI_rev	atgcaGGATCCgccgggaatgaccgcgagc<br>atgcaGCTAGCtcgaaacgctggcgggttcg	Amplification of full-length WT ponB from chromosome for pAV13 and pAV15
ponB-M46L_FW<br>ponB-M46L_rev	tgaagaaccgTTGccgcgcaaag<br>tcctcatagtcatcataatcgtcgtaatcatcgtcatc	Site directed mutagenesis for $^{\alpha}$PBP1b(M46L) point mutant in pAV13 and pAV15
XbaI_RBS-ponB-alpha-FW<br>ponB_rev_PstI	tacgaTCTAGAtttaagaaggagatatacatatgg ccgggaatgaccgcgagccaa<br>tttgcCTGCAGctcctgacgcaccag	Amplification of ponB-alpha from pAV13 for the generation of pAV27
ponB-alpha-R6E<br>pAV15_rev<br>pAV27_rev<br>pLM01_rev	cgggaatgacGAAgagccaattg<br>gcggatcctttgtagagctcatccatg<br>gccatatgtatatctccttcttaaatctagaggggaattg<br>gcggatccggaaatctccagagtag	Site directed mutagenesis for $^{\alpha}$PBP1b(R6E) point mutant in pAV35 (with pAV15_rev), pAV39 (pAV27_rev) and pLM001 (pLM01_rev)
$^{\text{N-pep }\alpha}$PBP1b-FW<br>$^{\text{N-pep }\alpha}$PBP1b-rev	atgcaTCTAGAaggagatatacatatggccggga atgaccgc<br>AAGCTTagcctctaccccgcgaaatac	Amplification of ponB amino acid 1-63 from GeneBlock with XbaI and HindIII restriction sites for pAV48.
$^{\text{N-pep }\gamma}$PBP1b-FW	atgcaTCTAGAaggagatatacatatgccgcgca aaggtaagg	Amplification of ponB gamma isoform (46-63). To be used with $^{\text{N-pep }\alpha}$PBP1b-rev for pAV49
$^{\text{N-pep }\alpha}$PBP1bR6E-FW	atgcaTCTAGAaggagatatacatatggccggga atgacGAAgag	Amplification of ponB alpha R6E isoform (1-63). To be used with $^{\text{N-pep }\alpha}$PBP1b-rev for pAV50

[a] mutated residues are capitalized. Restriction enzyme sites are capitalized and underlined.

Table S5. Synthetic DNA fragments used for POLAR assay.

Construct[a]	Nucleotide sequence[b]
$^{\text{N-pep}}$ $^{\alpha}$PBP1b(1-63)-msfGFP-H3H4	ctagaaggagatatacatatggccgggaatgaccgcgagccaattggacgcaaagggaaaccgacgcgtccggtcaaacaaaaggtaagcc<br>gtcgtcgttacgaagatgacgatgattacgacgattatgatgactatgaggatgaagaaccgttgccgcgcaaaggtaagggcaaaggcaaagg<br>gcgtaagcctcgtggcaaacgcggttctggcggtggatccaagggagaagagttgtttacgggtgttgtaccgattcttgtcgaactggacggagatg<br>tgaatggacataaattttcggtccgtggcgagggagagggtgacgctaccaacggaaaactcactcttaagttcatctgtaccaccggcaagctgcc<br>tgtcccatggccaacccttgtgacgactctgacttatggcgtgcaatgtttttcgcggtatcccgaccacatgaaacagcatgattttttcaagtcggcga<br>tgccagaaggatacgtgcaagagcggacgatctccttcaaggatgacggcacctacaagacccgtgccgaagttaagtttgagggcgatactttg<br>gtcaatcggattgagctgaaaggaatcgatttcaaagaagacggcaatatcctgggacacaagctcgaatataattttaacagccacaatgtatata<br>ttactgcggataaacagaagaatggcatcaaggcaaactttaaaattcgccataacgtagaagatggatcggtccaactcgccgaccactaccag<br>cagaacactccaatcggagatggcccggttttgcttccagataaccattatctcagcactcaatcaaagctcagcaaagaccccaacgagaagcg<br>ggatcacatggtactgttggagttcgtaaccgctgccggtatcacgcacggtatggacgaattgtataaaggaggtggttctagtgcgttattaatgcct<br>aaggatggccgcacacttgaagatgttgttagagaacttctgcgtcctttgcttaaagaatggcttgatcaaaatttgcctcgcattgttgagacgaaagt<br>ggaagaagaggttcaacgtatttcgcggggtagaggctaa
$^{\text{N-pep}}$ $^{\gamma}$PBP1b(46-63)-msfGFP-H3H4	ctagaaggagatatacatatgccgcgcaaaggtaagggcaaaggcaaagggcgtaagcctcgtggcaaacgcggttctggcggtggatccaag<br>ggagaagagttgtttacgggtgttgtaccgattcttgtcgaactggacggagatgtgaatggacataaattttcggtccgtggcgagggagagggtga<br>cgctaccaacggaaaactcactcttaagttcatctgtaccaccggcaagctgcctgtcccatggccaacccttgtgacgactctgacttatggcgtgca<br>atgtttttcgcggtatcccgaccacatgaaacagcatgattttttcaagtcggcgatgccagaaggatacgtgcaagagcggacgatctccttcaagg<br>atgacggcacctacaagacccgtgccgaagttaagtttgagggcgatactttggtcaatcggattgagctgaaaggaatcgatttcaaagaagacg<br>gcaatatcctgggacacaagctcgaatataattttaacagccacaatgtatatattactgcggataaacagaagaatggcatcaaggcaaactttaa<br>aattcgccataacgtagaagatggatcggtccaactcgccgaccactaccagcagaacactccaatcggagatggcccggttttgcttccagataa<br>ccattatctcagcactcaatcaaagctcagcaaagaccccaacgagaagcgggatcacatggtactgttggagttcgtaaccgctgccggtatcac<br>gcacggtatggacgaattgtataaaggaggtggttctagtgcgttattaatgcctaaggatggccgcacacttgaagatgttgttagagaacttctgcgt<br>cctttgcttaaagaatggcttgatcaaaatttgcctcgcattgttgagacgaaagtggaagaagaggttcaacgtatttcgcggggtagaggctaa
$^{\text{N-pep}}$ $^{\alpha}$PBP1b(R6E, 1-63)-msfGFP-H3H4	ctagaaggagatatacatatggccgggaatgacgaagagccaattggacgcaaagggaaaccgacgcgtccggtcaaacaaaaggtaagcc<br>gtcgtcgttacgaagatgacgatgattacgacgattatgatgactatgaggatgaagaaccgttgccgcgcaaaggtaagggcaaaggcaaagg<br>gcgtaagcctcgtggcaaacgcggttctggcggtggatccaagggagaagagttgtttacgggtgttgtaccgattcttgtcgaactggacggagatg<br>tgaatggacataaattttcggtccgtggcgagggagagggtgacgctaccaacggaaaactcactcttaagttcatctgtaccaccggcaagctgcc<br>tgtcccatggccaacccttgtgacgactctgacttatggcgtgcaatgtttttcgcggtatcccgaccacatgaaacagcatgattttttcaagtcggcga<br>tgccagaaggatacgtgcaagagcggacgatctccttcaaggatgacggcacctacaagacccgtgccgaagttaagtttgagggcgatactttg<br>gtcaatcggattgagctgaaaggaatcgatttcaaagaagacggcaatatcctgggacacaagctcgaatataattttaacagccacaatgtatata<br>ttactgcggataaacagaagaatggcatcaaggcaaactttaaaattcgccataacgtagaagatggatcggtccaactcgccgaccactaccag<br>cagaacactccaatcggagatggcccggttttgcttccagataaccattatctcagcactcaatcaaagctcagcaaagaccccaacgagaagcg<br>ggatcacatggtactgttggagttcgtaaccgctgccggtatcacgcacggtatggacgaattgtataaaggaggtggttctagtgcgttattaatgcct<br>aaggatggccgcacacttgaagatgttgttagagaacttctgcgtcctttgcttaaagaatggcttgatcaaaatttgcctcgcattgttgagacgaaagt<br>ggaagaagaggttcaacgtatttcgcggggtagaggctaa

[a] Synthetic DNA fragments were ordered as GeneBlocks, from Integrated DNA Technologies.

[b] RBS, N-terminal PBP1b peptide, linker, msfGFP, H3H4 PopZ interaction domain

Table S6. SPT statistics obtained from SpotOn[43].

Construct	Condition	Total trajectories[a]	Bound fraction	D_{Free} (μm^2 sec^{-1})
Halo-αPBP1b	Septum	349	0.227	0.081
		485	0.223	0.084
		575	0.252	0.079
		461	0.227	0.075
		599	0.224	0.084
		1029	0.215	0.081
	Side Wall	2873	0.150	0.092
		8060	0.153	0.087
		5331	0.164	0.088
		4531	0.138	0.089
		5994	0.134	0.099
		8957	0.155	0.094
Halo-γPBP1b	Septum	981	0.183	0.086
		445	0.115	0.065
		699	0.172	0.073
		559	0.112	0.075
		676	0.137	0.076
		860	0.173	0.071
	Side Wall	6716	0.138	0.073
		2695	0.139	0.064
		3027	0.118	0.067
		4052	0.155	0.074
		3158	0.128	0.080
		3555	0.097	0.079
Halo-αPBP1b(R6E)	Septum	1031	0.114	0.085
		314	0.112	0.083
		388	0.202	0.087
		419	0.186	0.080
		996	0.130	0.087
		652	0.186	0.084
	Side Wall	6190	0.121	0.094
		1325	0.122	0.094
		2084	0.111	0.092
		3695	0.128	0.099
		4761	0.120	0.095
		3991	0.131	0.092

[a] SPT movies were recorded for 30 s total observation period at 20 Hz acquisition frame rate.

Table S7. UniProt accession number of tested proteins for *in silico* analyses.

Organism	Protein	UniProt accession number	Experiment[a,b,c]	Amino Acids[d]
Escherichia coli	PBP1a	P02918	IDD	1-850
	PBP1b	P02919	AF/MSA/IDD	1-63 / 1-844
	FtsA	P0ABH0	AF	1-420
	FtsZ	P0A9A6	AF	1-382
Bacillus subtilis	PBP1	P39793	MSA	1-914
Buchnera apicola	PBP1b	Q89AR2	MSA	1-741
Burkholderia sp. *8Y*	PBP1b	A0A653UI87	AF/MSA	1-65 / 1-853
	FtsA	A0A653YP82	AF	1-420
Citrobacter freundii	PBP1b	A0AAE7GQM2	AF/MSA	1-59 / 1-845
	FtsA	A0A7G2IJP2	AF	1-341
Dickeya dadantii	PBP1b	E0SBK6	AF/MSA	1-62 / 1-833
	FtsA	E0SG72	AF	1-418
Erwinia tracheiphila	PBP1b	A0A0M2KDQ3	AF/MSA	1-61 / 1-827
	FtsA	A0A0M2KAZ1	AF	1-418
Gilliamella apicola	PBP1b	A0A556RJU6	AF	1-34 / 1-790
	FtsA	A0A1B9JIG1	AF	1-417
Paracoccus kondratievae	PBP1b	A0AAD3RRX7	MSA	1-774
Pantoea coffeiphila	PBP1b	A0A2S9I4K4	AF/MSA	1-65 / 1-853
	FtsA	A0A2S9I4U7	AF	1-418
Pectobacterium odoriferum	PBP1b	A0A094RW12	AF/MSA	1-67 / 1-825
	FtsA	A0A094U1E4	AF	1-418
Proteus mirablis	PBP1b	B4EUE0	AF/MSA	1-20 / 1-770
	FtsA	B4F107	AF	1-418
Providencia rettgeri	PBP1b	A0A379FN74	AF/MSA	1-68 / 1-841
	FtsA	A0A1B8SPZ0	AF	1-418
Pseudomonas aeruginosa	PBP1a	Q07806	IDD	1-822
	PBP1b	G3XD31	IDD	1-774
Shewanella pneumatophori	PBP1b	A0A9X2CHH1	AF/MSA	1-38 / 1-787
	FtsA	A0A9X2CH02	AF/MSA	1-349
Staphylococcus aureus	PBP1	A0A385MK68	MSA	1-744
Streptococcus pneumoniae	PBP1b	Q7CRA4	MSA	1-821
Vibrio cholerae	PBP1b	Q9KUC0	AF/MSA	1-30 / 1-777
	FtsA	Q9KPH0	AF	1-420
Xanthomonas hortorum	PBP1a	A0A6V7CFQ1	IDD	1-808
	PBP1b	A0AA47ICM5	MSA/IDD	1-815
Xenorhabdus innexi	PBP1b	A0A1N6MTY7	MSA	1-827
Yersinia enterocolitica	PBP1b	A0A9P1V5P0	AF/MSA	1-66 / 1-829
	FtsA	A0A0E1NAS2	AF	1-418

[a] AlphaFold interaction predictions were carried out on ColabFold v1.5.5 webserver (https://colab.research.google.com/github/sokrypton/ColabFold/blob/main/AlphaFold2.ipynb) using default parameters with indicated N-IDD peptide sequence of PBP1b and full-length FtsA.

[b] MSA were carried out using Clustal Omega on the EMBL webserver (https://www.ebi.ac.uk/jdispatcher/msa/clustalo) using default parameters.

[c] IDD sequence prediction was calculated on PrDOS server (https://prdos.hgc.jp/cgi-bin/top.cgi) with a 1% false positivity rate cutoff.

[d] Amio acid sequence length of N-IDD peptide or full-length protein.

Video S1. *In situ* architecture of sPG in wild-type *E. coli*. Cryo-electron tomogram of wild-type *E. coli*. Time-lapse series were acquired with a rate of 7 fps in the compressed format m4v for visualization purposes. Green, cyan and magenta layers indicate segmented IM, PG and OM, respectively. Ribosomes are shown in yellow. Scale bars = 100 nm.

Video S2. *In situ* architecture of sPG in Δ*ponB E. coli*. Cryo-electron tomograms of Δ*ponB* cells. Details as for Video S1.

Video S3. *In situ* architecture of sPG in Δ*lpoB E. coli*. Cryo-electron tomograms of Δ*lpoB* cells. Details as for Video S1.

Video S4. *In situ* architecture of sPG in Δ*ponA E. coli*. Cryo-electron tomograms of Δ*ponA* cells. Details as for Video S1.

Video S5. Time-lapse video of *E. coli* cells subjected to osmotic oscillations. Indicated strains were imaged in a microfluidic flow cell (CellAsic) in presence of 1µM SytoxGreen or 1µM propidium iodide (*$^{\alpha}$ponB(R6E)*). Osmotic oscillations were performed by switching media from LB to 0.5xLB0N ten times over a 42min observation period. Images were acquired at 0.1 Hz. Individual phase (center) and fluorescence (right) channels are shown in addition to a merged overlay (left). Representative examples for WT, Δ*ponB*, Δ*ponB pNP146 (P_{ara}::sulA)*, Δ*ponB pAV39 (P_{lac}::ponB(R6E, M46L)),* Δ*lpoB, and* Δ*ponA* are sequentially shown. For further details, see *Methods.* Scale bar = 2 µm.

Video S6. Side-by-side comparison of SPT experiments with *E. coli* cells expressing Halo fusions to the indicated PBP1b isoforms. STP trajectories of indicated Halo-PBP1b isoforms (labeled with 20 nM JF549) are colored according to their mean track speed (blue = slow, red = fast). ZapA-sfGFP (false-colored in green) was used as a fiducial marker for the divisome and overlayed over a bright field reference image. Images were recorded at 20 Hz and binned 2x2. For further details, see *Methods.* Scale bar = 0.5 µm.

Video S7. FtsZ treadmilling assay in response to N-$^{\alpha}$PBP1b peptide addition. Alexa488 labeled FtsZ (1.25 µM) and unlabeled FtsA (0.4 µM) were reconstituted on supported lipid bilayers (SLBs) in the presence of 4 mM ATP/GTP and allowed to self-organize into treadmilling filaments and followed by TIRF microscopy at 0.5 Hz acquisition frame rate. At 2 min, 0.4 µM Cy5 labeled N-$^{\alpha}$PBP1b peptide was added to the reaction chamber. For further details, see *Methods.* Scale bar = 5 µm.

Video S8. FtsZ treadmilling assay in response to N-$^{\alpha}$PBP1b(R6E) peptide addition. Alexa488 labeled FtsZ (1.25 µM) and unlabeled FtsA (0.4 µM) were reconstituted on SLBs in presence of 4 mM ATP/GTP and allowed to self-organize into treadmilling filaments and followed by TIRF microscopy at 0.5 Hz acquisition frame rate. At 2 min, 0.4 µM Cy5 labled N-$^{\alpha}$PBP1b(R6E) peptide was added to the reaction chamber. For further details, see *Methods.* Scale bar = 5 µm.

Extended Data Table 1. Hits *from* AlphaFold multimer screen for PBP1b isoforms. For additional information check: https://private.predictomes.org/library/help.

REFERENCES

1. Navarro, P. P. *et al.* Cell wall synthesis and remodelling dynamics determine division site architecture and cell shape in Escherichia coli. *Nat Microbiol* **7**, 1621–1634 (2022).
2. Szwedziak, P., Wang, Q., Freund, S. M. V. & Löwe, J. FtsA forms actin-like protofilaments. *EMBO J* **31**, 2249–2260 (2012).
3. Paradis-Bleau, C. *et al.* Lipoprotein cofactors located in the outer membrane activate bacterial cell wall polymerases. *Cell* **143**, 1110–1120 (2010).
4. Eddy, S. R. A new generation of homology search tools based on probabilistic inference. *Genome Inform* **23**, 205–211 (2009).
5. Mendler, K. *et al.* AnnoTree: visualization and exploration of a functionally annotated microbial tree of life. *Nucleic Acids Research* **47**, 4442–4448 (2019).
6. Kremer, J. R., Mastronarde, D. N. & McIntosh, J. R. Computer visualization of three-dimensional image data using IMOD. *J. Struct. Biol.* **116**, 71–76 (1996).
7. Mastronarde, D. N. & Held, S. R. Automated tilt series alignment and tomographic reconstruction in IMOD. *J. Struct. Biol.* **197**, 102–113 (2017).
8. Coray, R., Navarro, P., Scaramuzza, S., Stahlberg, H. & Castaño-Díez, D. Automated fiducial-based alignment of cryo-electron tomography tilt series in Dynamo. *Structure* **32**, 1808-1819.e4 (2024).
9. Buchholz, T.-O. *et al.* Content-aware image restoration for electron microscopy. *Methods Cell Biol* **152**, 277–289 (2019).
10. Lamm, L. *et al.* MemBrain: A deep learning-aided pipeline for detection of membrane proteins in Cryo-electron tomograms. *Comput Methods Programs Biomed* **224**, 106990 (2022).
11. Lamm, L. *et al.* MemBrain v2: an end-to-end tool for the analysis of membranes in cryo-electron tomography. 2024.01.05.574336 Preprint at https://doi.org/10.1101/2024.01.05.574336 (2024).
12. Ermel, U. H., Arghittu, S. M. & Frangakis, A. S. ArtiaX: An electron tomography toolbox for the interactive handling of sub-tomograms in UCSF ChimeraX. *Protein Sci* **31**, e4472 (2022).
13. Meng, E. C. *et al.* UCSF ChimeraX: Tools for structure building and analysis. *Protein Science* **32**, e4792 (2023).
14. Bernhardt, T. G. & De Boer, P. A. J. Screening for synthetic lethal mutants in Escherichia coli and identification of EnvC (YibP) as a periplasmic septal ring factor with murein hydrolase activity. *Molecular Microbiology* **52**, 1255–1269 (2004).
15. Cho, H. *et al.* Bacterial cell wall biogenesis is mediated by SEDS and PBP polymerase families functioning semi-autonomously. *Nat Microbiol* **1**, 1–8 (2016).

16. Peters, N. T., Dinh, T. & Bernhardt, T. G. A Fail-Safe Mechanism in the Septal Ring Assembly Pathway Generated by the Sequential Recruitment of Cell Separation Amidases and Their Activators. *Journal of Bacteriology* **193**, 4973–4983 (2011).
17. de Lorenzo, V. & Timmis, K. N. Analysis and construction of stable phenotypes in gram-negative bacteria with Tn5- and Tn10-derived minitransposons. *Methods Enzymol* **235**, 386–405 (1994).
18. Buss, J. A., Peters, N. T., Xiao, J. & Bernhardt, T. G. ZapA and ZapB form an FtsZ-independent structure at midcell. *Mol Microbiol* **104**, 652–663 (2017).
19. Lim, H. C. & Bernhardt, T. G. A PopZ-linked apical recruitment assay for studying protein-protein interactions in the bacterial cell envelope. *Mol Microbiol* **112**, 1757–1768 (2019).
20. Cherepanov, P. P. & Wackernagel, W. Gene disruption in Escherichia coli: TcR and KmR cassettes with the option of Flp-catalyzed excision of the antibiotic-resistance determinant. *Gene* **158**, 9–14 (1995).
