# Supplementary material for: The penicillin-binding protein PBP1b fortifies the Escherichia coli division site against osmotic rupture
Source: Nat Microbiol. 2026 Jul 3;11(8):2142–56. doi: 10.1038/s41564-026-02403-6 (PMC13423804; doi:10.1038/s41564-026-02403-6)
Supplement: Supplementary file 2 — Reporting Summary [file 41564_2026_2403_MOESM2_ESM.pdf]

nature portfolio

Corresponding author(s): Thomas G. Bernhardt; Paula P. Navarro

Last updated by author(s): Apr 14, 2025

Reporting Summary

Nature Portfolio wishes to improve the reproducibility of the work that we publish. This form provides structure for consistency and transparency in reporting. For further information on Nature Portfolio policies, see our Editorial Policies and the Editorial Policy Checklist.

Statistics

For all statistical analyses, confirm that the following items are present in the figure legend, table legend, main text, or Methods section.

n/a	Confirmed	
☐	☒	The exact sample size (n) for each experimental group/condition, given as a discrete number and unit of measurement
☐	☒	A statement on whether measurements were taken from distinct samples or whether the same sample was measured repeatedly
☐	☒	The statistical test(s) used AND whether they are one- or two-sided<br>*Only common tests should be described solely by name; describe more complex techniques in the Methods section.*
☐	☒	A description of all covariates tested
☐	☒	A description of any assumptions or corrections, such as tests of normality and adjustment for multiple comparisons
☐	☒	A full description of the statistical parameters including central tendency (e.g. means) or other basic estimates (e.g. regression coefficient) AND variation (e.g. standard deviation) or associated estimates of uncertainty (e.g. confidence intervals)
☐	☒	For null hypothesis testing, the test statistic (e.g. F, t, r) with confidence intervals, effect sizes, degrees of freedom and P value noted<br>*Give P values as exact values whenever suitable.*
☒	☐	For Bayesian analysis, information on the choice of priors and Markov chain Monte Carlo settings
☒	☐	For hierarchical and complex designs, identification of the appropriate level for tests and full reporting of outcomes
☐	☒	Estimates of effect sizes (e.g. Cohen's d, Pearson's r), indicating how they were calculated

Our web collection on statistics for biologists contains articles on many of the points above.

Software and code

Policy information about availability of computer code

Data collection	SerialEM 3.2 (open source, cited) and Thermo Fisher Scientific Tomography v5.3.0 (Thermo Fisher Scientific) for operation of electron microscopes (references in Methods).<br>Light microscopy data was acquired using Nikon Elements 5.1 (references in Method).<br>Atomic force microscopy data was acquired using JPK acquisition software v6.4
Data analysis	Cryo-ET data processing (all available and referenced in Methods); IMOD v4.11.4 (open source, cited), Amira-Avizon 2023.2 (Thermo Fisher Scientific), Dynamo 1.1.532 (open source, cited), MATLAB 2023b (MathWorks).<br>Light Microscopy: FIJI (open source, cited). All particle tracking was done with the Trackmate plugin within FIJI (open source, cited), then analyzed with SpotOn (open source, cited). MicrobeJ (cited, open source) and JPK data processing software were used for image segmentation and AFM analysis.

For manuscripts utilizing custom algorithms or software that are central to the research but not yet described in published literature, software must be made available to editors and reviewers. We strongly encourage code deposition in a community repository (e.g. GitHub). See the Nature Portfolio guidelines for submitting code & software for further information.

Data

Policy information about availability of data

All manuscripts must include a data availability statement. This statement should provide the following information, where applicable:

- Accession codes, unique identifiers, or web links for publicly available datasets
- A description of any restrictions on data availability
- For clinical datasets or third party data, please ensure that the statement adheres to our policy

Cryo-ET data: representative tomograms (binned 8) are deposited in EMDB with accession codes: EMD-27479 (wild-type), EMD-53351 (ΔponB), EMD-53357 (ΔlpoB), EMD-53363 (ΔponA). Corresponding raw movie frames are uploaded to EMPIAR and will be release upon acceptance of the manuscript: EMPIAR-11090 (wild-type), EMPIAR-13502 (ΔponB), EMPIAR-13513 (ΔlpoB) and EMPIAR-13491 (ΔponA).
Light microscopy, AFM data and growth curves are uploded to Zenodo: https://doi.org/10.1101/2025.04.02.646830

Research involving human participants, their data, or biological material

Policy information about studies with human participants or human data. See also policy information about sex, gender (identity/presentation), and sexual orientation and race, ethnicity and racism.

Reporting on sex and gender	*Use the terms sex (biological attribute) and gender (shaped by social and cultural circumstances) carefully in order to avoid confusing both terms. Indicate if findings apply to only one sex or gender; describe whether sex and gender were considered in study design; whether sex and/or gender was determined based on self-reporting or assigned and methods used. Provide in the source data disaggregated sex and gender data, where this information has been collected, and if consent has been obtained for sharing of individual-level data; provide overall numbers in this Reporting Summary. Please state if this information has not been collected. Report sex- and gender-based analyses where performed, justify reasons for lack of sex- and gender-based analysis.*
Reporting on race, ethnicity, or other socially relevant groupings	*Please specify the socially constructed or socially relevant categorization variable(s) used in your manuscript and explain why they were used. Please note that such variables should not be used as proxies for other socially constructed/relevant variables (for example, race or ethnicity should not be used as a proxy for socioeconomic status). Provide clear definitions of the relevant terms used, how they were provided (by the participants/respondents, the researchers, or third parties), and the method(s) used to classify people into the different categories (e.g. self-report, census or administrative data, social media data, etc.) Please provide details about how you controlled for confounding variables in your analyses.*
Population characteristics	*Describe the covariate-relevant population characteristics of the human research participants (e.g. age, genotypic information, past and current diagnosis and treatment categories). If you filled out the behavioural & social sciences study design questions and have nothing to add here, write "See above."*
Recruitment	*Describe how participants were recruited. Outline any potential self-selection bias or other biases that may be present and how these are likely to impact results.*
Ethics oversight	*Identify the organization(s) that approved the study protocol.*

Note that full information on the approval of the study protocol must also be provided in the manuscript.

Field-specific reporting

Please select the one below that is the best fit for your research. If you are not sure, read the appropriate sections before making your selection.

☒ Life sciences ☐ Behavioural & social sciences ☐ Ecological, evolutionary & environmental sciences

For a reference copy of the document with all sections, see nature.com/documents/nr-reporting-summary-flat.pdf

Life sciences study design

All studies must disclose on these points even when the disclosure is negative.

Sample size	Sample sizes were determined by available cryo-electron microscopy and cryo-FIB instrument time. A total of 60 tomograms were acquired (see Methods and Extended Data). For light microscopy experiments no specific sample size calculation were made since a large number of cells were analyzed (500-1000 cells for each experiment and strain). AFM sample sizes were determined by available scope time. A total of 113 sacculi were imaged and 46 at high-resolution.
Data exclusions	For cryo-ET, tilt-series exhibiting errors during data collection were excluded. Exclusions of error-containing or incomplete tilt-series is a standard practice for cryo-ET data processing. For light microscopy SPT detection outside of cells were rejected as described in Methods. For AFM analysis regions of folded peptidoglycan were excluded from analysis.
Replication	Tomogram were acquired from different cells, different lamellae and different grids (at least 3 grids per strain, except for ΔponA were only 1

Replication	grid could be imaged due to physical damage of the grid). Cells were vitrified on different days and from different batch cultures.<br>All light microscopy experiments were successfully repeated over three biological replicates.
Randomization	No randomization of data was performed. All data collection and data analysis were performed in the same manner (see Methods). Cells for cryo-FIB milling and tilt-series collection were chosen randomly on the grid.<br>For light microscopy, samples were imaged in randome intervals. For live-cell imaging, cells one field of view away from the edge of the agarose pad were imaged.
Blinding	It was not possible to blind any of our data during acquisition. For segmentation of tomograms, this task was performed by three different people that did not know the genotype of the imaged strains. For determination of cell lysis sites data was analyzed by three different people that did not know the genotype of the imaged strain. For the rest of experiments and data analysis no blinding was performed.

Reporting for specific materials, systems and methods

We require information from authors about some types of materials, experimental systems and methods used in many studies. Here, indicate whether each material, system or method listed is relevant to your study. If you are not sure if a list item applies to your research, read the appropriate section before selecting a response.

Materials & experimental systems

n/a	Involved in the study	
☐	☒	Antibodies
☒	☐	Eukaryotic cell lines
☒	☐	Palaeontology and archaeology
☒	☐	Animals and other organisms
☒	☐	Clinical data
☒	☐	Dual use research of concern
☒	☐	Plants

Methods

n/a	Involved in the study	
☒	☐	ChIP-seq
☒	☐	Flow cytometry
☒	☐	MRI-based neuroimaging

Antibodies

Antibodies used	Custom polyclonal rabbit serum against PBP1b (Paradis-Bleau et al., 2010)
Validation	Antibody was validated by Paradis-Bleau et al., 2010. It does not cross react with a ΔponB strain.

Plants

Seed stocks	-
Novel plant genotypes	-
Authentication	-
